# Neurotrophic effects of G_M1_ ganglioside, NGF, and FGF2 on canine dorsal root ganglia neurons *in vitro*

**DOI:** 10.1038/s41598-020-61852-z

**Published:** 2020-03-25

**Authors:** S. Schwarz, A. Lehmbecker, W. Tongtako, K. Hahn, Y. Wang, F. Felmy, I. Zdora, G. Brogden, K. Branitzki-Heinemann, M. von Köckritz-Blickwede, W. Baumgärtner, I. Gerhauser

**Affiliations:** 1 Department of Pathology, University of Veterinary Medicine Hannover, Bünteweg 17, 30559 Hannover, Germany; 2 Center of Systems Neuroscience, Hannover, Germany; 3 c/o Faculty of Veterinary Science, Prince of Songkla University, Hat Yai, Thailand; 4 Institute of Zoology, University of Veterinary Medicine Hannover, Bünteweg 17, 30559 Hannover, Germany; 5 Department of Physiological Chemistry, University of Veterinary Medicine Hannover, Bünteweg 17, 30559 Hannover, Germany; 6 Research Center for Emerging Infections and Zoonoses (RIZ), University of Veterinary Medicine Hannover, Bünteweg 17, 30559 Hannover, Germany

**Keywords:** Cell death, Cell death, Cell growth, Cell growth

## Abstract

Dogs share many chronic morbidities with humans and thus represent a powerful model for translational research. In comparison to rodents, the canine ganglioside metabolism more closely resembles the human one. Gangliosides are components of the cell plasma membrane playing a role in neuronal development, intercellular communication and cellular differentiation. The present *in vitro* study aimed to characterize structural and functional changes induced by G_M1_ ganglioside (G_M1_) in canine dorsal root ganglia (DRG) neurons and interactions of G_M1_ with nerve growth factor (NGF) and fibroblast growth factor (FGF2) using immunofluorescence for several cellular proteins including neurofilaments, synaptophysin, and cleaved caspase 3, transmission electron microscopy, and electrophysiology. G_M1_ supplementation resulted in increased neurite outgrowth and neuronal survival. This was also observed in DRG neurons challenged with hypoxia mimicking neurodegenerative conditions due to disruptions of energy homeostasis. Immunofluorescence indicated an impact of G_M1_ on neurofilament phosphorylation, axonal transport, and synaptogenesis. An increased number of multivesicular bodies in G_M1_ treated neurons suggested metabolic changes. Electrophysiological changes induced by G_M1_ indicated an increased neuronal excitability. Summarized, G_M1_ has neurotrophic and neuroprotective effects on canine DRG neurons and induces functional changes. However, further studies are needed to clarify the therapeutic value of gangliosides in neurodegenerative diseases.

## Introduction

Laboratory rodents are used frequently in research but recent studies recommended the companion dog as an additional model to understand spontaneous diseases of the nervous system like spinal cord injury and inflammatory diseases of the central nervous system (CNS)^1,2^ as well as the biological and environmental factors that influence aging and neurodegenerative processes^3–6^. Dogs have already been used to evaluate surgical techniques and novel medications due to strong similarities between human and canine diseases, treatments, and response to therapy^2,4,7–13^. Furthermore, canine neurons have previously been used as a model to study canine distemper virus infection, a paramyxovirus closely related to the human measles virus^14,15^. The structure and sequence of the human genome is also more similar to the dog than to the mouse genome^16,17^ and almost 60% of canine hereditary diseases represent potential models for human diseases (http://omia.angis.org.au/home/; February 05, 2020).

There is evidence that application of monosialotetrahexosylganglioside (G_M1_) has therapeutic potential, for example in the treatment of acute spinal cord injury^18,19^. In addition, clinical trials demonstrated that G_M1_ has symptomatic and disease modifying effects on different human neurodegenerative diseases^20–22^. Several studies in rodents have demonstrated that the neuroprotective effect of G_M1_ seems to be dependent on the interaction with certain growth factors^23^. Gangliosides are sialic acid-containing glycosphingolipids that can be found in different cellular membranes. In the CNS gangliosides account for up to 10% of the total lipid content in neurons^24^. They are involved in intercellular communication, cellular differentiation, neuronal development, and regeneration^25–28^ due to their capacity to modulate Ca^2+^ influx and their influence on the function of receptors for muscarinic acetylcholine, serotonin, glutamine, neurotransmitters, and neurotrophic factors^29^. Cellular gangliosides concentrate in lipid microdomains termed lipid rafts, which are signaling platforms rich in cholesterol and glycosphingolipids and contain amongst others high affinity tropomyosin-related kinase (Trk) and low affinity neurotrophin receptors (p75^NTR^)^30–32^. These receptors are activated by nerve growth factor (NGF) and other neurotrophic factors and have a strong impact on neuronal development, maintenance, and survival as well as memory formation and storage^33,34^. Anti-G_M1_ antibodies modulate the membrane-associated sphingomyelin metabolism by altering neutral sphingomyelinase activity thereby inhibiting NGF action on rat pheochromocytoma (PC12) cells^35,36^. G_M1_ and NGF protect primary cultured rat embryonic dorsal root ganglia (DRG) and spinal cord neurons from glutamate-induced excitotoxicity^37,38^. Both molecules may function by modulating Ca^2+^ homeostasis, maintaining normal mitochondrial membrane potential or by promoting the mRNA expression of neuronal proteins such as growth associated protein 43 and neurofilaments (NFs)^38,39^. G_M1_ also protects rats against high altitude cerebral edema by suppressing oxidative stress and production of the pro-inflammatory cytokines IL-1β, IL-6 and TNF-α^40^. Nevertheless, another study using an ischemia/reperfusion model in rats demonstrated that G_M1_ neuroprotection seems to be dependent on p75^NTR^ ^41^.

Exogenous gangliosides also directly interact with growth factors such as basic fibroblast growth factor (FGF2) and inhibit FGF receptor binding^42,43^. Moreover, G_M1_ can inhibit FGF2-mediated effects by acting on the same intracellular signaling molecules. For instance, FGF2 stimulates the activity of glycogen synthase kinase 3 (GSK3) in proliferating adult rat hippocampal progenitor cells, whereas G_M1_ prevents GSK3 activation in organotypic hippocampal slice cultures^44,45^. In contrast, cell-associated G_M1_ has been described to act as a functional co-receptor for FGF2^43^. Despite its name, FGF2 plays an essential role in neurogenesis, differentiation, axonal branching, and neuron survival in various types of brain and peripheral nerve lesions^46,47^. FGF2 also stimulates the proliferation of neuronal precursor cells isolated from postnatal murine DRGs^48^. The ability of adult rat ganglion cells to regrow axons *in vitro* can be influenced by FGF2 and G_M1_^49^. Neurotrophic effects of FGF2 seem to be also mediated by soluble mediators released from glial cells^50^. Summarized, NGF, FGF2 and gangliosides such as G_M1_ can exert neurotrophic effects, which might be exploited in the treatment of traumatic and degenerative diseases of the nervous system. In addition, the recent development of semisynthetic and potent G_M1_ compounds as well as solid lipid nanoparticles as drug delivery systems motivates the therapeutic use of G_M1_^51,52^.

G_M1_ levels in rodent brains increase with age^53^, whereas an age-associated decrease in G_M1_ content has been demonstrated in the human brain^54^. Moreover, G_M1_ can be metabolized by an alternative asialo degradation pathway in mice but not in humans^55^ and dogs^56^. In general, humans and dogs seem to have a similar G_M1_ metabolism^57–59^ indicating that dogs represent an appropriate animal model to characterize synergistic neurotrophic effects of gangliosides in human neuronal cells. Nevertheless, a detailed *in vitro* study in canine neurons analyzing the influence of G_M1_ on neurite outgrowth, axonal transport systems, and neuronal functions including interactions with the neurotrophic growth factors NGF and FGF2 has not yet been performed. Consequently, the aim of the present study was to characterize neurotrophic effects of G_M1_, NGF, and FGF2 with special emphasis on cytoskeletal protein expression and electrophysiological changes in canine DRG neurons as suitable translational approach^2,10,60^. In order to observe the effect of G_M1_ in a model mimicking neurodegenerative conditions due to disruptions of energy homeostasis occurring in pathological conditions such as traumatic spinal cord injury^61^, neurons were additionally cultured in a hypoxia glove-chamber (1% O_2_) with and without G_M1_ supplementation.

## Results

### NGF and G_M1_ ganglioside induce the formation of neurites

**Figure 1:**
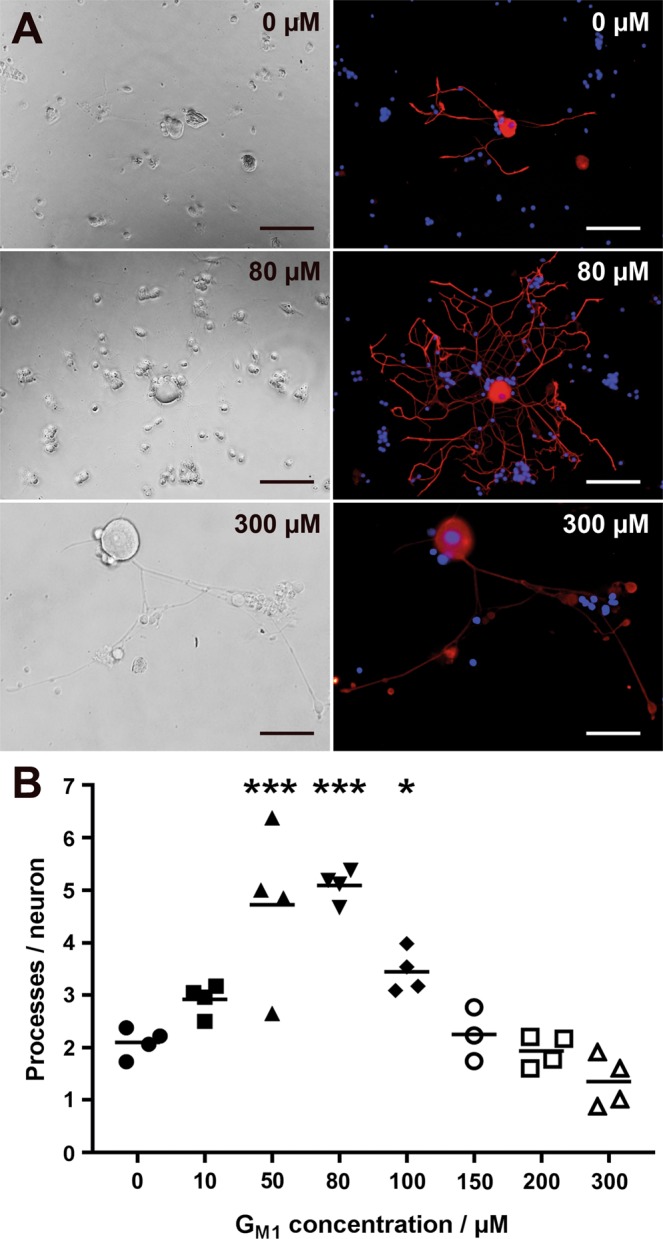
Ganglioside titration in adult canine dorsal root ganglia neurons. (**A**) Phase contrast and immunofluorescence pictures of neurons incubated with 0, 80, and 300 µM G_M1_ ganglioside and stained for neuronal class III β tubulin 2 days post seeding. Bars = 100 µm. (**B**) The graph shows the number of immunopositive processes per neuron for each G_M1_ ganglioside concentration (single values of four dogs with means). *P < 0.05; ***P < 0.001.

Neurons grown without supplements (Sato’s medium with 1% BSA) showed a mean number of 2.1 βIII tubulin-positive processes at 2 days post seeding (dps). In order to study dose-dependent effects of G_M1_ on neurite outgrowth, neurons were incubated with increasing ganglioside concentrations. The mean number of processes per neuron significantly increased at a G_M1_ ganglioside concentration of 50 µM (4.7), 80 µM (5.2), and 100 µM (3.4) (Fig. 1). A ganglioside concentration of 80 µM was used for all further sets of *in vitro* experiments.

**Figure 2:**
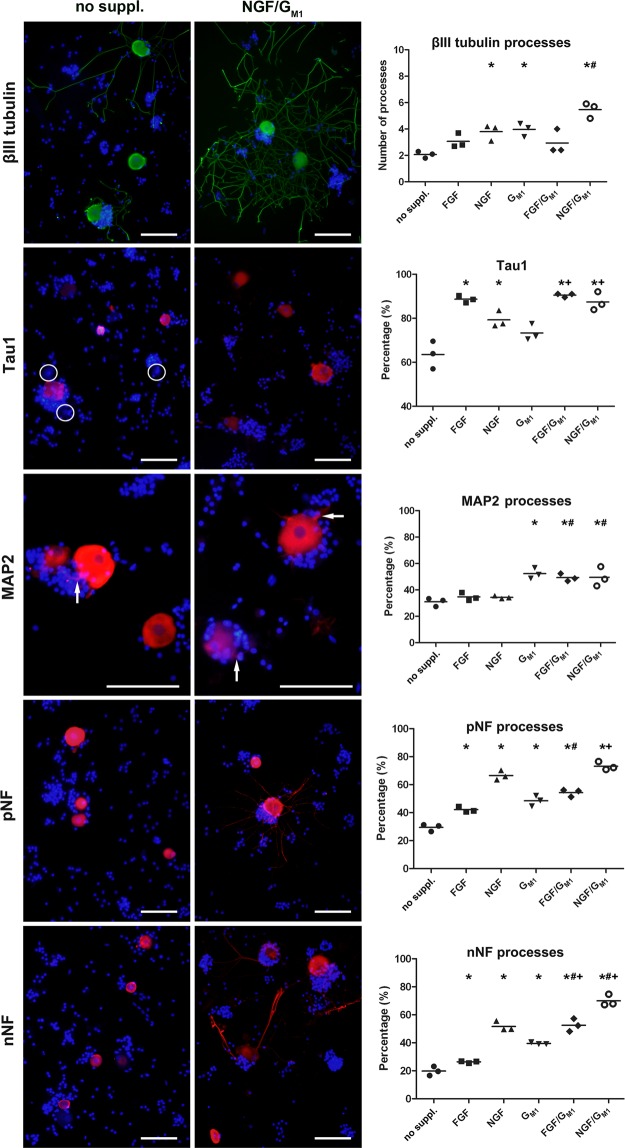
Neuronal class III β tubulin, Tau1, MAP2, phosphorylated neurofilament (pNF), and non-phosphorylated neurofilament (nNF) expression in canine dorsal root ganglia neurons. The neurons were grown without growth factors or G_M1_ ganglioside (no supplements) or supplemented with fibroblast growth factor 2 (FGF), nerve growth factor (NGF), G_M1_, FGF and GM_1_, or NGF and G_M1_ and stained 2 days post seeding. Shown are representative pictures of neurons cultured without supplements and neurons supplemented with NGF and G_M1_. Encircled areas mark Tau1 negative neurons. Note MAP2 positive processes of neurons (arrows). Bars = 100 µm. The graphs show the number of βIII tubulin positive processes per neuron, the percentage of Tau1 positive neurons, and the percentages of neurons with processes immunopositive for MAP2, pNF, and nNF for each condition (single values of 3 dogs with means). *Statistically significant differences (*P* < 0.05) compared to neurons cultured without supplements. ^#^Statistically significant differences (*P* < 0.05) of FGF/G_M1_ and NGF/G_M1_ compared to FGF and NGF, respectively. ^+^Statistically significant differences (*P* < 0.05) of FGF/G_M1_ and NGF/G_M1_ compared to G_M1_ only. No suppl. = no supplements.

In order to analyze further growth promoting factors, the neurotrophic properties of NGF and FGF2 alone or in combination with G_M1_ ganglioside were analyzed (Fig. 2; Suppl. Fig. 1). Culture of DRG neurons without NGF resulted in a significantly decreased mean number of βIII tubulin-positive processes per neuron (2.1 processes per neuron) when compared to neurons cultured with NGF (3.8 processes per neuron). Supplementation of neurons with G_M1_ ganglioside lead to a significant increase in the mean number of processes in cultures both with and without NGF supplementation (5.5 and 3.9 processes per neuron, respectively). Furthermore, neurons supplemented with both NGF and G_M1_ possessed significantly more processes than neurons cultured with FGF2 alone (3.1) or a combination of FGF2 and G_M1_ (2.9) In contrast, G_M2_ and G_M3_ supplementation did not affect neurite outgrowth and the glucosylceramide synthase inhibitor D-PDMP blocked neurite outgrowth (Suppl. Figs. 2 and 3). Microtubules formed by tubular polymers of tubulins represent an important part of the cytoskeleton, which is known to be affected by neurotrophin signaling pathways^62–64^. Consequently, the observed effects of G_M1_ ganglioside, NGF, and FGF2 on DRG neurons instigated the analysis of their impact on additional components of the neuronal cytoskeleton focusing on microtubular proteins and NFs.

### G_M1_ and growth factors differentially affect the neuronal cytoskeleton

NFs and microtubule-associated proteins (MAPs) such as Tau1 and MAP2 represent major components of the neuronal cytoskeleton. The percentage of neurons with Tau1-positive somata significantly increased from 63% in neurons cultured without supplements (Sato’ medium with 1% BSA only) to 79% and 89% after supplementation with NGF and FGF2, respectively. Significantly more Tau1-positive cells were also found in NGF/G_M1_ ganglioside (87%) and FGF2/G_M1_ ganglioside (91%) containing media but G_M1_ supplementation alone (73%) did not have a significant influence on neuronal Tau1 expression (Fig. 2; Suppl. Figure 1). These results demonstrated that NGF and FGF2 but not G_M1_ ganglioside supplementation affect Tau1 expression. Consequently, we also investigated the effect of these neurotrophic factors on MAP2 expression and NF phosphorylation status using antibodies directed against non-phosphorylated NF (nNF) and phosphorylated NF (pNF).

The percentage of cells with MAP2-positive processes was significantly higher in culture conditions containing G_M1_ ganglioside (G_M1_: 52%; FGF2/G_M1_: 49%; NGF/G_M1_: 50%) than in culture conditions without G_M1_ (control: 31%; FGF2: 35%; NGF: 34%), whereas addition of NGF or FGF2 alone did not change MAP2 expression in neuronal processes (Fig. 2). 20% and 29% of neurons cultured without supplements demonstrated nNF- and pNF-positive processes, respectively (Fig. 2; Suppl. Figure 1). There was an increase in this percentage after FGF2 (nNF: 26%; pNF: 42%), NGF (nNF: 52%; pNF: 67%), G_M1_ (nNF: 40%; pNF: 48%), and FGF2/G_M1_ supplementation (nNF: 53%; pNF: 54%). The highest percentage of cells with nNF- or pNF-positive processes was found after NGF/G_M1_ supplementation (nNF: 70%; pNF: 73%), which actually significantly exceeded supplementation with NGF or G_M1_ alone. Interestingly, the pNF/nNF ratio decreased from 1.45 (29%/20%) in neurons cultured without supplements to 1.04 (73%/70%) in neurons supplemented with NGF and G_M1_. These results indicate that the phosphorylation status of neurofilaments is affected by a combination of NGF and G_M1_ ganglioside. The changes in the neuronal cytoskeleton might affect axonal transport mechanisms and synaptic plasticity^65,66^. Therefore, the expression of the cytoskeletal motor proteins dynein and kinesin and synaptophysin, a synaptic vesicle glycoprotein was investigated.

### NGF and G_M1_ ganglioside stimulate the formation of synaptophysin accumulations in neuronal processes and disturb axonal transport mechanisms

**Figure 3:**
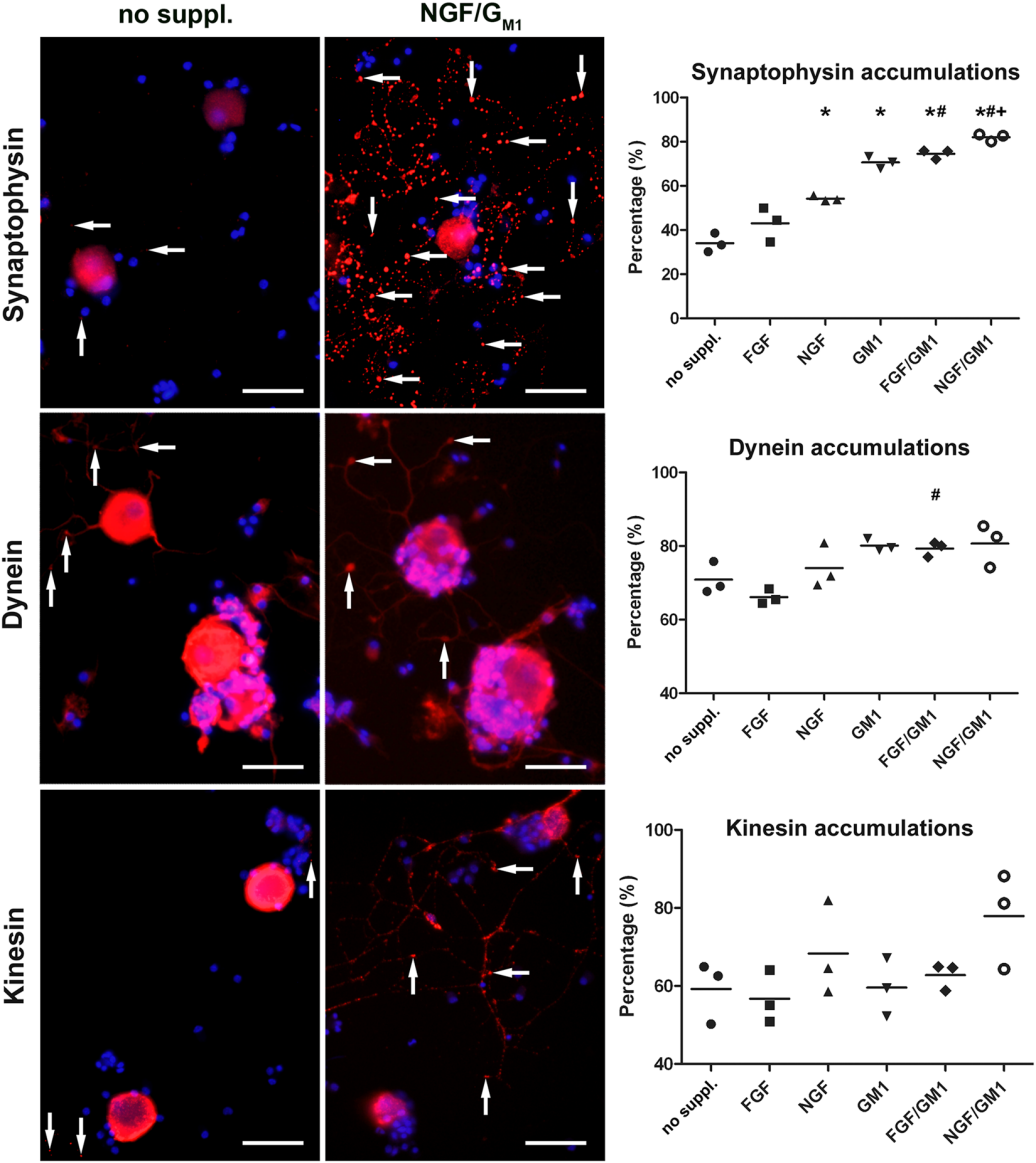
Synaptophysin, dynein, and kinesin expression in canine dorsal root ganglia neurons. The neurons were grown without growth factors or G_M1_ ganglioside (no supplements) or supplemented with fibroblast growth factor 2 (FGF), nerve growth factor (NGF), G_M1_, FGF and GM_1_, or NGF and G_M1_ and stained 2 days post seeding. Shown are representative pictures of neurons cultured without supplements and neurons supplemented with NGF and G_M1_. Note accumulations of synaptophysin, dynein, and kinesin most likely within neuronal processes (arrows). Bars = 100 µm. The graphs show the percentages of neurons with processes containing respective accumulations for each condition (single values of 3 dogs with means). *Statistically significant differences (*P* < 0.05) compared to neurons cultured without supplements. ^**#**^Statistically significant differences (*P* < 0.05) of FGF/G_M1_ and NGF/G_M1_ compared to FGF and NGF, respectively. ^+^Statistically significant differences (*P* < 0.05) of FGF/G_M1_ and NGF/G_M1_ compared to G_M1_ only. No suppl. = no supplements.

**Figure 4:**
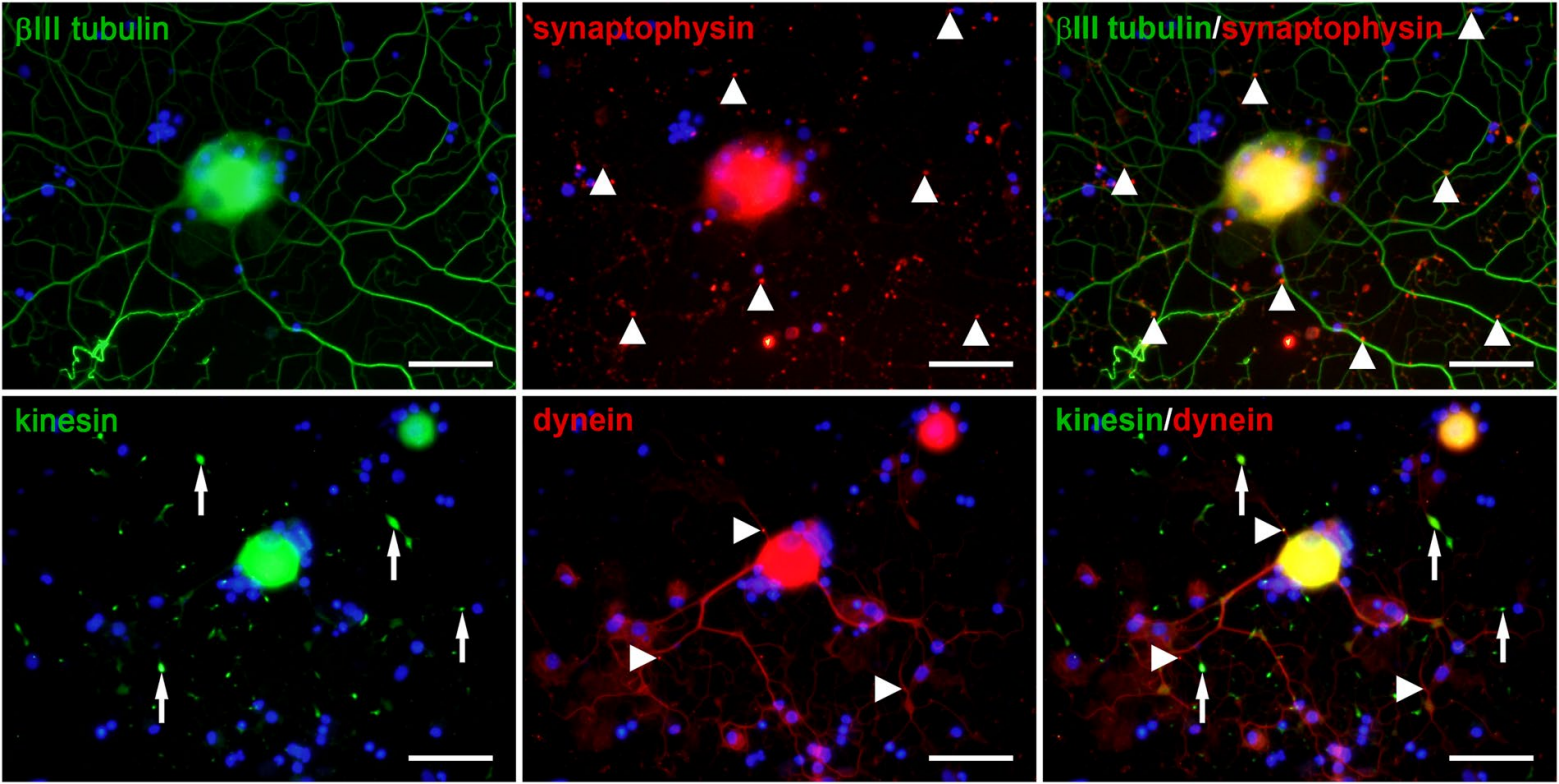
Immunofluorescence double-labeling of adult canine dorsal root ganglia neurons. Neurons were supplemented with NGF and G_M1_ and stained 2 days post seeding for neuronal class III β tubulin (green) and synaptophysin (red) or kinesin (green) and dynein (red). Note synaptophysin and dynein accumulations (arrowheads) as well as kinesin accumulations (arrows) in neuronal processes. Bars, 50 μm.

Accumulations of synaptophysin, dynein, and kinesin in neuronal processes were observed in all culture conditions (Figs. 3, 4; Suppl. Fig. 4). 34% of neurons cultured without supplements demonstrated synaptophysin accumulations in their neurites. There was a significant increase in this percentage after addition of NGF (54%), G_M1_ (71%), FGF2/G_M1_ (75%), and NGF/G_M1_ (82%). FGF2 alone (43%) did not change the percentage of synaptophysin accumulations in neuronal processes (Fig. 3; Suppl. Fig. 4). Moreover, dynein and kinesin accumulations were observed in neurites of 72% and 59% of neurons cultured without supplements, respectively (Fig. 4). G_M1_ ganglioside and growth factor supplementation did not have a significant impact on the presence of accumulations of dynein or kinesin in neurites compared to untreated conditions (Fig. 3; Suppl. Fig. 4). Nevertheless, there was a significant increase in the percentage of neurons with dynein accumulations in their processes after G_M1_ supplementation (G_M1_: 80%; FGF2/G_M1_: 80%; NGF/G_M1_: 80%) compared to the addition of FGF2 alone (67%). Moreover, the highest percentage of neurons with kinesin accumulations (78%) was observed after NGF/G_M1_ supplementation. These results indicate that a combination of NGF and G_M1_ ganglioside affects the distribution of the synaptic vesicle protein synaptophysin as well as anterograde and retrograde axonal transport mechanisms. These transport mechanisms are also critically involved in trafficking of neurotrophin receptor such as p75^NTR^ ^67^, which prompted the analysis of p75^NTR^ expression.

### G_M1_, NGF, and FGF2 synergistically induce the internalization and/or down-regulation of p75^NTR^

**Figure 5:**
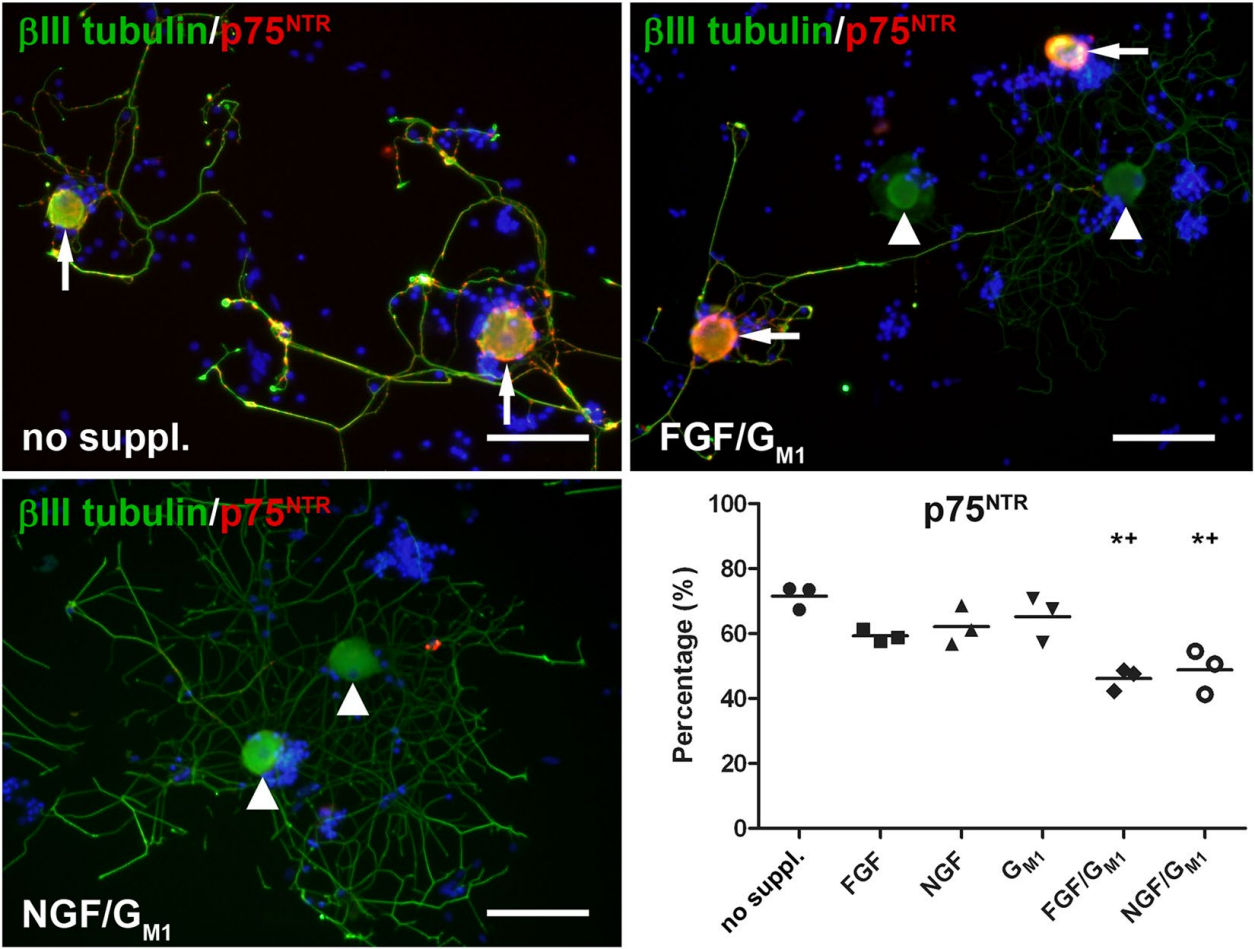
Expression of the p75 neurotrophin receptor (p75^NTR^) in canine dorsal root ganglia neurons. The neurons were grown without growth factors or G_M1_ ganglioside (no supplements) or supplemented with fibroblast growth factor 2 (FGF), nerve growth factor (NGF), G_M1_, FGF and GM_1_, or NGF and G_M1_ and stained for neuronal class III β tubulin (green) and p75^NTR^ (red) 2 days post seeding. Shown are representative pictures of neurons cultured without supplements and neurons supplemented with FGF and G_M1_ as well as NGF and G_M1_. Note p75^NTR^ positive neurons (arrows) and p75^NTR^ negative neurons (arrowheads). Bars = 100 µm. The graph shows the percentage of p75^NTR^ positive neurons for each condition (single values of 3 dogs with means). *Statistically significant differences (*P* < 0.05) compared to neurons cultured without supplements. ^+^Statistically significant differences (*P* < 0.05) of FGF/G_M1_ and NGF/G_M1_ compared to G_M1_ only. No suppl. = no supplements.

The p75^NTR^ expression was significantly higher in neurons supplemented with FGF2/G_M1_ (46%) or NGF/G_M1_ (49%) compared to neurons cultured without supplements (72%), whereas addition of FGF2 (59%), NGF (62%), or G_M1_ ganglioside (65%) alone had no significant effect (Fig. 5; Suppl. Fig. 5). Consequently, only the combination of G_M1_ ganglioside and growth factors affected the expression of p75^NTR^, which binds all neurotrophins including NGF.

Depending on its interactions with co-receptors, p75^NTR^ can induce apoptosis in DRG neurons or generate survival signals^68–71^. The impact of G_M1_ ganglioside and growth factors on cell survival was investigated by studying cleaved caspase 3 expression, which plays a central role in the execution phase of apoptosis.

### G_M1_, NGF and FGF2 favor neuronal survival

**Figure 6:**
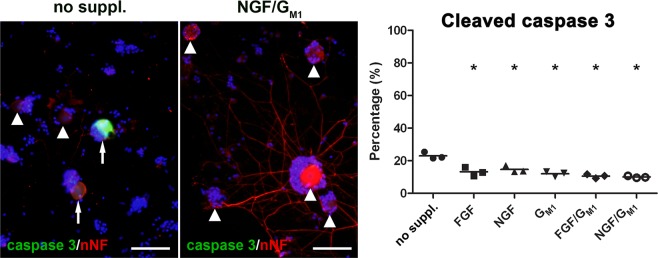
Cleaved caspase 3 expression in canine dorsal root ganglia neurons. The neurons were grown without growth factors or G_M1_ ganglioside (no supplements) or supplemented with fibroblast growth factor 2 (FGF), nerve growth factor (NGF), G_M1_, FGF and GM_1_, or NGF and G_M1_ and stained for cleaved caspase 3 (green) and non-phosphorylated neurofilament (nNF, red) 2 days post seeding. Shown are representative pictures of neurons cultured without supplements and neurons supplemented with NGF and G_M1_. Note cleaved caspase 3 positive neurons (arrows) and cleaved caspase 3 negative neurons (arrowheads). Bars = 100 µm. The graph shows the percentage of cleaved caspase 3 positive neurons for each condition (single values of 3 dogs with means). *Statistically significant differences (*P* < 0.05) compared to neurons cultured without supplements. No suppl. = no supplements.

A significant decrease in the number of neurons expressing cleaved caspase 3 was present in neurons supplemented with G_M1_, NGF and FGF2 (FGF2: 13%; NGF: 15%; G_M1_: 12%; FGF2/G_M1_: 11%; NGF/G_M1_: 10%) compared to neurons cultured without supplements (23%) underlining their neurotrophic activity and neuroprotective function (Fig. 6; Suppl. Fig. 6). In contrast, G_M2_ and G_M3_ supplementation had no significant effect on the survival of canine DRG neurons (Suppl. Figs. 2 and 3). Depletion of gangliosides by D-PDMP induced neuronal cell death as detected by a Trypan blue dye exclusion assay (Suppl. Fig. 3).

### Exogenously administered G_M1_ accumulates in non-raft membrane fractions of neuroblastoma cells

Lipid raft isolation and analysis of primary DRG neurons did not reveal concentrations of G_M1_ above the limit of detection due to limited numbers of isolated cells (data not shown). Therefore, N1E-115 murine neuroblastoma cells were used to determine the cellular localization of exogenously administered G_M1_. Separation of glycolipids on a thin layer chromatography (TLC) plate revealed the presence of G_M1_ in non-raft (NR) membrane fractions in G_M1_ treated cells (Suppl. Fig. 7). Confirmation of the presence of G_M1_ in the NR fraction was determined through the addition of 500 ng G_M1_ to the same sample. A clear increase in the intensity of the band at the same height of the standard was seen when compared to the untreated NR G_M1_ treated sample. The difference between band intensities corresponds to the addition of 500 ng G_M1_. G_M1_ could not be detected in the untreated cells nor the lipid raft (LR) fraction from the G_M1_ treated cells, indicating that the amount present is below the limit of detection of 50 ng. Furthermore, G_M1_ could be detected in the medium containing 80 µM of G_M1_ and not in the medium without the addition of G_M1_.

Summarized, the results of the immunofluorescence revealed an impact of G_M1_, NGF and FGF2 on neurite formation, the neuronal cytoskeleton, synaptophysin expression, axonal transport mechanisms and neuronal survival. The prominent contribution of G_M1_ ganglioside to these effects instigated additional investigations on ultrastructural and electrophysiological changes induced by G_M1_ ganglioside.

### G_M1_ supplementation of neurons leads to an increased density of cytoplasmic multivesicular bodies

**Figure 7:**
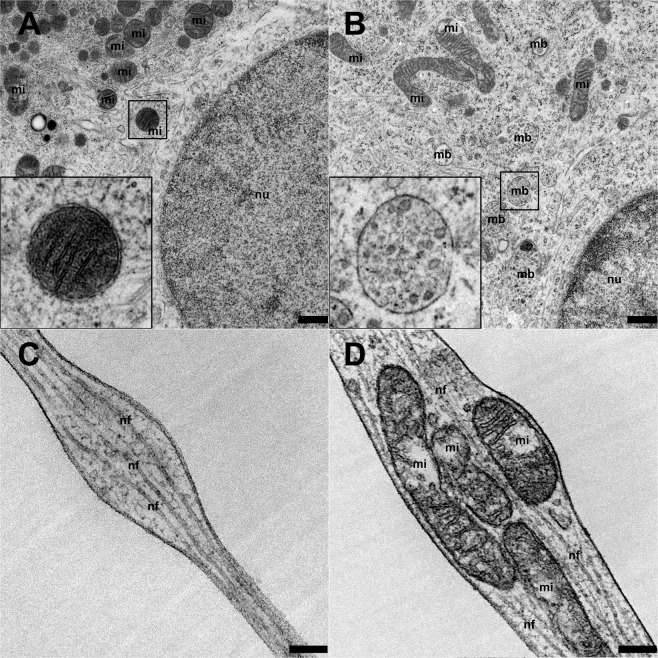
Transmission electron microscopy of cultivated canine dorsal root ganglia neurons. Higher numbers of cytoplasmic multivesicular bodies were found in neurons supplemented with 80 µM G_M1_ ganglioside **(B)** compared to controls **(A**, 0 µM G_M1_**)**. Inserts: Higher magnification of a mitochondrium (mi) and a multivesicular body (mb). There were nodular enlargements of neurites with neurofilaments **(C**, 0 µM G_M1_**)** and mitochondria **(D**, 80 µM G_M1_**)** under both conditions. Bars, 0.5 µm (**A,B**), 0.2 µm (**C,D**), nf, neurofilaments; nu, nucleus.

Neurons supplemented with 80 µM G_M1_ ganglioside displayed a higher density of cytoplasmic multivesicular bodies compared to neurons cultured without supplements (0 µM G_M1_: 0.07/µm^2^; 80 µM G_M1_: 0.28/µm^2^; *P* < 0.0001; Fig. 7). Nodular enlargements of neurites containing accumulations of mitochondria, small vesicular structures, and/or neurofilaments were found in neurons with and without G_M1_ ganglioside supplementation (0 µM G_M1_: 0.176/µm; 80 µM G_M1_: 0.155/µm; Fig. 7). The percentage of nodular enlargements containing mitochondria increased from 24% (5 nodular enlargements with mitochondria/21 total number of nodular enlargements) in neurites of neurons cultured without supplements to 35% (13/37) in neurites of neurons supplemented with 80 µM G_M1_. However, this increase was not statistically significant (*P* = 0.5557).

### G_M1_ supplementation leads to an elevated resting potential, a reduced action potential current threshold, and a slowing down of depolarization speed

**Figure 8:**
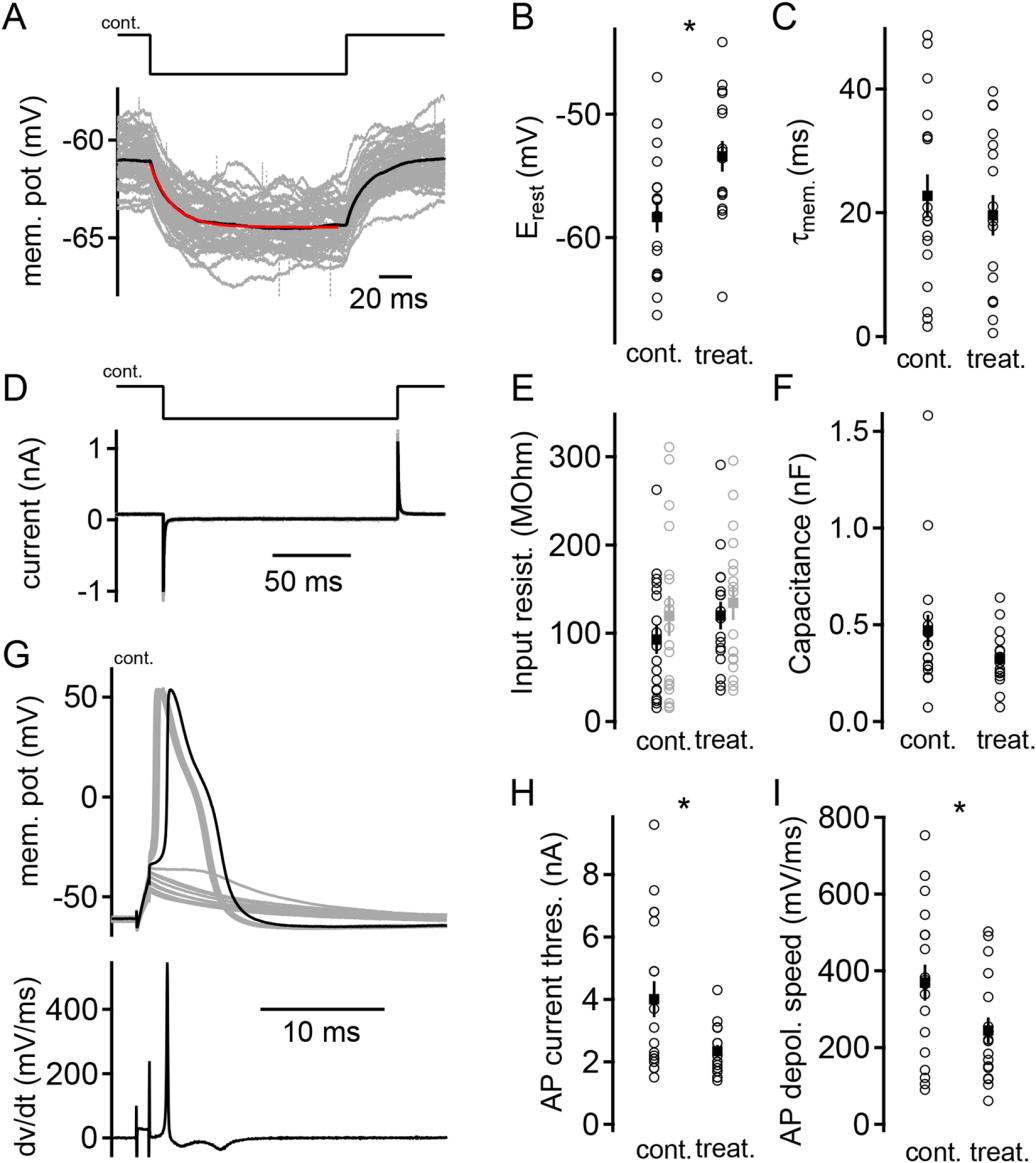
Functional characterization of G_M1_ treatment on large dorsal root ganglia (DRG) neurons isolated from adult dogs *in vitro*. (**A**) Single (gray) and average (black) voltage deflections to a −25 pA hyperpolarization current injection (top) to a non-treated (control) DRG neuron. An exponential fit to the decay of the hyperpolarization is indicated by the red line. (**B**) Resting potential of non-treated (control) and G_M1_ supplemented DRG neurons. Individual cells are shown by open circles, whereas averages are indicated by filled squares. A T-test demonstrated a significant difference with a value of *P* = 0.008; control: n = 18; treatment n = 17. Symbols and number of n are identical throughout this figure. (**C**) Membrane decay time constants calculated from exponential fits shown in (**A**). (**D**) Membrane current and capacitive transients to a −10 mV hyperpolarization of a non-treated (control) DRG neuron. Single response is illustrated in gray, average in black. (**E**) Membrane input resistance of non-treated (control) and G_M1_ supplemented DRG neurons. Black symbols display data calculated from current clamp recordings depicted in (**A**), gray symbols from voltage clamp recordings exemplified in (**D**). Both recording configurations were performed in the same set of cells. Ohm’s law was used to compute input resistance. (**F**) Membrane capacitance of large DRG neurons calculated from voltage clamped current transients shown in (**D**). To account for the full charging of the cell’s membrane the time taken into account was determined by using a bi-exponential weighted decay time constant which was multiplied by three. (**G**) Sub- and supra-threshold responses of a non-treated (control) DRG neuron to 1 ms current injections. Black trace shows first supra-threshold response used to analyze action potential properties including dv/dt analysis depicted at the bottom. (**H**) Action potential current threshold was demonstrated to be significantly different between non-treated (control) and G_M1_ supplemented DRG neurons (T-test: *P* = 0.011). (**I**) Speed of the rise of action potentials (dv/dt in mV/ms). A significant (T-test: *P* = 0.04) slower depolarization speed was found in G_M1_ supplemented compared to non-treated (control) DRG neurons.

To probe whether ganglioside supplementation affects the functional properties of cultured canine large DRG neurons, the membrane properties at rest were quantified and the action potential generation was analyzed. From three dogs 18 neurons cultured in Sato’s medium with NGF only and 17 neurons additionally treated with 80 µM G_M1_ were recorded by analyzing one well-plate without and one with G_M1_ treatment from each dog. Resting membrane properties, such as resting potential, input resistance, membrane time constant and effective cell capacity were extracted from a small 120 ms long hyperpolarization^68,69^ induced by a −25 pA current injection (Fig. 8A). The resting potential was determined before the hyperpolarization and was significantly different (*P* = 0.008) between the non-treated (−58.3 ± 1.2 mV) and G_M1_-treated (−53.4 ± 1.2 mV) DRG neurons (Fig. 8B). The membrane time constant, measured from a mono-exponential fit to the onset of the hyperpolarization (Fig. 8A) remained unaffected by the treatment (non-treated: 22.7 ± 0.3 ms; G_M1_-treated: 19.5 ± 0.3 ms; *P* = 0.514; Fig. 8C). The input resistance was calculated according to Ohm’s law (Fig. 7A,D). In current and in voltage clamp the input resistance was unchanged between non-treated (current clamp: 119.7 ± 22.6 MOhm; voltage clamp: 92.9 ± 16.2 MOhm) and G_M1_-treated cells (current clamp: 134.5 ± 19.3 MOhm; voltage clamp: 119.9 ± 15.6 MOhm; current clamp *P* = 0.624; voltage clamp *P* = 0.24; Fig. 8E). The effective capacitance, the membrane surface that can be charged from the soma was on average 471.2 ± 80.8 pF for non-treated cells and 324.9 ± 34.2 pF for G_M1_-treated cells (*P* = 0.112; Fig. 8F).

Action potential properties can be measured from the first supra-threshold response to a 1 ms long current injection incremented with 100 pA (Fig. 8G)^70^. In addition to the recorded voltage wave form, its differentiation was quantified in order to gain insights into the speed of de- and repolarizations (Fig. 8G). Only two action potential parameters indicated a significant difference between non-treated and G_M1_-treated DRG neurons. The average action potential current threshold was significantly lowered by the treatment (non-treated: 4.01 ± 0.58 nA; G_M1_-treated: 2.34 ± 0.2 nA; *P* = 0.0119; Fig. 8H). The speed of depolarization was significantly slowed down by the treatment (non-treated: 368.9 ± 46.8 mV/ms; G_M1_-treated: 244.5 ± 33.9; *P* = 0.0407; Fig. 8I). All other action potential parameters, such as voltage threshold (non-treated: −29.6 ± 1.2 mV; G_M1_-treated: −26.1 ± 1.6 mV), height (non-treated: 114.6 ± 3.0 mV; G_M1_-treated: 108.6 ± 3.5 mV), width from voltage threshold (non-treated: 2.7 ± 0.3 ms; G_M1_-treated: 2.4 ± 0.3 ms), the size of after hyperpolarization (non-treated: −9.8 ± 1.2 mV; G_M1_-treated: −9.9 ± 1.1 mV), and speed of repolarization (non-treated: −46.6 ± 4.2 mV/ms; G_M1_-treated: −41.1 ± 4.2 mV/ms) remained unaffected in the treated cells.

### G_M1_ supplementation results in increased neurite outgrowth and viability in neurons cultured under hypoxic conditions

**Figure 9:**
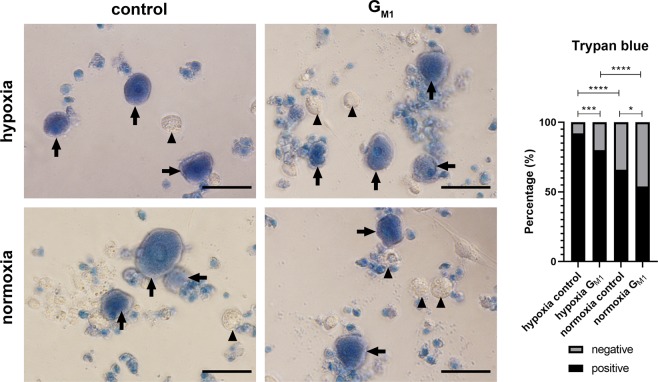
Trypan blue dye exclusion assay: Influence of G_M1_ treatment upon viability of neurons cultured under hypoxic and normoxic conditions. Neurons were grown in Sato’s medium containing NGF under hypoxia (1% O_2_) or normoxia (21% O_2_) with and without G_M1_ treatment for 6 days. Culture under hypoxic conditions lead to an overall decreased viability, while G_M1_ treatment was able to significantly increase the viability of neurons cultured both under hypoxic and normoxic conditions. Shown are representative images of neurons cultured under hypoxia and normoxia with (G_M1_) and without (control) G_M1_ treatment. Note positive/dead (arrows) and negative/viable neurons (arrowheads). Bars = 50 μm. The graph shows the percentages of negative and positive cells for each condition. *P < 0.05; ***P < 0.001; ****P < 0.0001.

When neurons were cultured under hypoxia (1% O_2_) for 6 days directly after seeding in Sato’s medium containing NGF, the viability as determined by Trypan blue dye exclusion assay was significantly reduced (7.9%) when compared to neurons cultured under normoxia (33.6%). Treatment with 80 µM G_M1_ resulted in a significant increase in viability in neurons both cultured under hypoxia and normoxia (hypoxia G_M1_-treated: 20.0%; normoxia G_M1_-treated: 46.1%) when compared to neurons cultured in Sato’s medium containing NGF only (control). Cultures supplemented with G_M1_ under hypoxia showed a significantly lower viability than G_M1_-treated neurons cultured under normoxia (Fig. 9). There were no significant differences in cleaved caspase 3 expression between the groups of this experiment (hypoxia control: 9.67%; hypoxia G_M1_: 8.40%; normoxia control: 16.02%; normoxia G_M1_: 4.28%; Suppl. Fig. 8).

**Figure 10:**
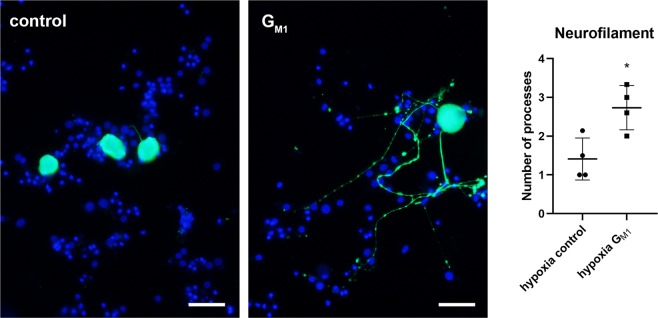
Influence of G_M1_ treatment upon number of processes of neurons cultured for 6 days under normoxia followed by 48 hours of hypoxia: Neurons were cultured for 6 days under normoxia (21% O_2_) followed by a 48-hour period of hypoxia (1% O_2_), during which they either remained in Sato’s medium supplemented with NGF (control) or received G_M1_ treatment (G_M1_). G_M1_ treatment under hypoxic conditions lead to a significant increase in number of processes per neuron. Neurons are stained with an antibody against pan-neurofilament. Bars = 50 µm. The graph shows the number of neurofilament positive processes per neuron (single values of four wells with means). *P < 0.05.

As neurons cultured under hypoxia for 6 days directly after seeding showed an almost complete lack of neurite-outgrowth, it was not possible to determine the impact of G_M1_ treatment upon neurite-outgrowth under hypoxic conditions. Therefore, a second set of experiments was performed. Neurons were first cultured under normoxia for 6 days and afterwards transferred to the hypoxia glove-chamber for 48 hours. G_M1_ treatment during the hypoxia-period of 48 hours resulted in a significant increase in mean neurite-number per neuron (2.7 neurites/neuron) as compared to control neurons cultured in Sato’s medium containing NGF only (1.4 neurites per neuron; Fig. 10). When culturing neurons for 6 days under normoxia followed by 48 hours of hypoxia, there was no significant impact of G_M1_ treatment upon the viability as determined by Trypan blue dye exclusion assay (control: 55,47%; G_M1_: 53.06%; Suppl. Fig. 9). Cleaved caspase 3 expression was slightly reduced in hypoxic neurons treated with G_M1_ (35.95%) compared to control neurons (39.77%), albeit it did not reach significance (Suppl. Fig. 10).

## Discussion

There is accumulating evidence of a beneficial effect of G_M1_ application upon different pathological conditions of the nervous system, like spinal cord injury, high altitude cerebral edema, and neurodegenerative diseases^19,40,72^. Furthermore, age-dependent alterations in the amount and distribution of gangliosides in the CNS seem to be involved in the pathogenesis of neurodegenerative diseases due to their impact on neuronal maintenance, stability, and regeneration^28,53,73–75^. However, effects of ganglioside application on neuronal protein expression and especially functional activities have not been investigated in a species with a ganglioside metabolism similar to humans so far. The present study revealed that G_M1_, but not G_M2_ and G_M3_ ganglioside has neurotrophic effects on canine DRG neurons such as inducing neurite outgrowth and suppressing apoptosis. Interestingly, even though neurodegenerative changes induced by NGF withdrawal in rodent neurons are mostly reported to affect embryonal neurons^76–79^, the culture of DRG neurons from adult dogs without NGF supplementation in the present study lead to a significantly reduced number of neurites, an increased apoptotic rate and decreased synaptophysin-accumulations. Therefore, in contrast to previous studies focusing on rodent neurons, it seems that NGF withdrawal continues to have a negative impact on canine DRG neurons cultured *in vitro* during later stages of development. This negative effect of NGF withdrawal was more than compensated by the supplementation with G_M1_, while the combined treatment with both NGF and G_M1_ appeared to be even more beneficial. In contrast, FGF2 supplementation only caused minor changes. Intriguingly, viability and neurite outgrowth of canine DRG neurons subjected to hypoxia (1% O_2_), occurring in different pathological conditions, was likewise ameliorated by G_M1_ supplementation.

Moreover, G_M1_ ganglioside supplementation affected the neuronal cytoskeleton including the phosphorylation status of NFs, synaptophysin expression, axonal transport mechanisms, cell metabolism, and biophysical properties. A previous study described that gangliosides increased the expression of NF proteins in embryonic chicken DRG neurons^80^. G_M1_ also potentiated NGF activity on neurite outgrowth and NF expression in rat PC12 cells^81^. In addition, NGF induced an increase in neuronal processes in adult rat DRG neurons *in vitro* and promoted neurite length and arborization in bovine DRG neurons^82,83^. Likewise, G_M1_ triggered an increased neurite outgrowth in canine DRG neurons characterized by higher numbers of neuronal processes positive for βIII tubulin and NFs. G_M1_ also enhanced MAP2 expression in these processes, which might be related to neurite initiation^84^. No effect of G_M1_ was found on Tau1 expression, whereas NGF and FGF2 induced its expression in canine DRG neurons. FGF2 also induced Tau1 expression in proliferating adult rat hippocampal progenitor cells^45^. Anti-apoptotic effects of G_M1_, NGF, and also FGF2 on canine DRG neurons were substantiated by a decreased neuronal cleaved caspase 3 expression. Similarly, pro-survival effects were also described for NGF^85^ and G_M1_^86^ in rodents, whereas apoptosis was induced by FGF2 in murine DRG neurons after sciatic nerve injury^87^. G_M1_ also counteracted lead-induced apoptosis by decreasing the expression of Bax and cleaved caspase 3 and by increasing the level of Bcl-2 in the developing rat hippocampus^88^.

The present lipid analysis showed an increase in G_M1_ in the non-raft (NR) fraction in G_M1_ treated cells compared to the NR fraction of non-treated cells. Similarly, exogenous G_M1_ appears to be predominately partitioned into NR fractions in rat cerebellar granule cells^89^, whereas G_M1_ has been shown to primarily be localized within plasma membrane lipid rafts under normal conditions^90^. This could be the result of normal cellular trafficking pathways being bypassed leading to NR localization. However, this localization does not seem to prevent the influence of G_M1_ on neurotrophin interactions with their receptors. A current study indicated that despite belonging to separate membrane domains TrkA might interact with G_M1_ by laying down its extracellular portion onto the membrane, thereby approaching the oligosaccharide portion of G_M1_^91^.

Recent studies investigated the molecular basis of the interactions between G_M1_ and neurotrophins including NGF and brain-derived neurotrophic factor (BDNF)^91,92^. G_M1_ acts as a bridge able to increase and stabilize the interactions of NGF with its high affinity receptor TrkA, which promotes neurite formation in murine neuroblastoma (Neuro2a) cells^92^. G_M1_ also induces the release of BDNF from hippocampal neurons^93^ and the amount of G_M1_ in the environment of the BDNF receptor TrkB can modulate its activity^94^. Increased interactions between neurotrophins and their receptors in response to G_M1_ supplementation likely stimulate several intracellular signaling pathways explaining the observed neurotrophic effects. Trk receptors activate the phosphatidylinositol-3 kinase (PI3K)-protein kinase B (AKT), RAS-mitogen-activated protein kinase (MAPK) and phospholipase C (PLC)-γ-protein kinase C (PKC) pathways^64,95^. The PI3K-AKT pathway has antiapoptotic activity and controls dendritic arborization together with the RAS-MAPK pathway^96–98^. The RAS-MAPK signaling cascade promotes neuronal differentiation including neurite outgrowth^64^. Extracellular signal-regulated kinase (ERK) and p38 are two MAPKs, which are activated by NGF to stimulate the phosphorylation of the cAMP response element-binding protein (CREB) and influence gene transcription^99,100^. For instance, BDNF regulates dendritic branching in a CREB-dependent manner by increasing the expression of the guanine deaminase cypin, which directly binds to tubulin heterodimers and promotes microtubule polymerization and formation of proximal dendrites^62,101^. Complexes of BDNF and TrkB also activate small GTPases of the Rho family including Cdc42/Rac/RhoA to stimulate actin and microtubule synthesis and growth of neuronal fibers^62^. Furthermore, activation of PLC-γ by BDNF triggers Ca^2+^- and PKC-regulated pathways that promote synaptic plasticity^62–64,102^. BDNF not only modulates the number of synapses by regulating the morphology of the axonal tree but also promotes synapse formation and maintenance *in vivo*^69,103^. Moreover, TrkB signaling mediates survival of hippocampal and motor neurons after axotomy^104^. Neurotrophins including their precursor forms such as pro-BDNF can also bind to p75^NTR^, which can modulate and even counteract neurotrophic effects mediated by Trk receptors^62^. This low affinity neurotrophin receptor acts on RhoA, JNK/c-jun and NF-κB signaling pathways, which promote neuronal growth cone development, apoptosis and, neuronal survival, respectively^63,64^. The PI3K-AKT, RAS-MAPK and PLC-γ-PKC pathways are also activated by FGF2, which can bind to four different signaling tyrosine kinase FGF receptors mediating FGF family functions in embryogenesis and organogenesis as well as in metabolism, tissue repair, and regeneration^105^. In the CNS, FGF signaling is involved in neuronal differentiation, migration and excitability, synaptogenesis, myelination and learning and memory formation^105^.

In addition to the described neurotrophic effects, supplementation of canine DRG neurons with G_M1_ and NGF amplified accumulations of synaptophysin in their processes. This effect is possibly related to an increase in endocytosis and/or retrograde transport of synaptic vesicles or anterograde transport of synaptophysin molecules. The transfer of synaptophysin from the soma to neurites can be induced by NGF in cultured newborn rat trigeminal ganglion neurons, which promotes activity-dependent maturation of synaptic connections^106^. The present synaptophysin accumulations might also be linked to an increased synapse formation, which could be further enhanced by the increased number of neuronal processes resulting in an enlarged contact area of the DRG neurons. Similarly, previous studies demonstrated that gangliosides have an impact on the synaptic plasticity and function of rat hippocampal neurons and thereby influence long term potentiation and learning ability^107^. Formation and maintenance of synapses are dependent on various retrograde messengers including neurotrophins^108^. Consequently, an increased binding, internalization, and retrograde transport of NGF receptors in signaling endosomes might promote synaptogenesis^109–111^. Similarly, the stimulation of canine DRG neurons with NGF and FGF2 most likely results in an internalization of p75^NTR^ proteins due to ligand binding and thereby in a decrease of p75^NTR^ expression on the cell surface. In addition, G_M1_ augmented the formation of dynein accumulations in canine DRG neurons indicating changes in axonal transport such as increased retrograde transport. Likewise, the transport rate of tubulin and actin in sensory fibers of adult Sprague-Dawley rats can be stimulated by ganglioside treatment, which could be caused by molecular interactions between cytoskeletal elements and integral membrane glycolipids^112^.

Interestingly, one study described accumulations of synaptophysin as a reliable immunohistochemical marker for axonal damage in CNS lesions^113^. Moreover, the present study demonstrated that G_M1_ and NGF supplementation results in an increased expression of pNF and nNF within neuronal processes, which was accompanied by a decrease in the pNF/nNF ratio possibly reflecting changes in the NF phosphorylation status. Normal axons are characterized by highly phosphorylated NFs, whereas damaged axons found in different inflammatory, degenerative and traumatic CNS diseases^114–116^ and disturbed axonal transport^117,118^ contain nNFs. Thus, supplementation of canine DRG neurons with G_M1_ and NGF might also cause degenerative changes in neuronal processes. To allow a more in-depth analysis of this effect, an ultrastructural analysis was performed revealing that G_M1_ induced the formation of cytoplasmic multivesicular bodies. These intracellular endosomal organelles are characterized by a single outer membrane containing a varying number of internal vesicles. They were initially described as prelysosomal structures but actually participate in various endocytic and trafficking functions, including protein sorting, recycling, transport, storage, and release, and represent a general indicator of neuronal stress^119^. The increased number of multivesicular bodies found in neurons from aged animals seems to be associated with age-related neurodegenerative changes^119^. Nevertheless, several studies also demonstrated that multivesicular bodies contain signaling molecules and their receptors allowing their recruitment for membrane insertion, which might affect synaptic plasticity^120–122^. Interestingly, multivesicular bodies located within the neuronal soma and dendrites accumulate NGF, whereas endosomes mediate the retrograde axonal transport of growth factors such as NGF^123^. Thus the G_M1_-dependent formation of multivesicular bodies in the soma of the present canine DRG neurons might represent a sign of neuronal stress and degenerative changes and/or result from an enhanced trafficking of membrane receptors such as p75^NTR^ ^124,125^.

To clarify the impact of the described morphological changes on functional properties of canine DRG neurons, an electrophysiological analysis was performed. G_M1_ supplementation induced an elevated resting potential, a reduced action potential current threshold, and a slowing down of the depolarization speed. The elevated membrane potential might have partially caused the reduction of the current threshold and possibly the speed of depolarization. The elevation of the membrane potential approximates the action potential threshold and thus less current might be needed to stimulate the neuron. Moreover, the depolarization entails an increased inactivation of sodium channels in DRG neurons^126,127^. This reduced availability of sodium channels might result in a slowing down of the action potential during the depolarization phase. However, considering the age-dependent decrease in G_M1_ content in the human brain the effects of G_M1_ supplementation on neuronal electrical excitability might be exploited to reduce impaired functional capacities of aged brains.

In summary, the present study showed neurotrophic and neuroprotective effects of G_M1_ gangliosides on DRG neurons isolated from adult dogs, which were characterized by increased neuronal survival and neurite outgrowth and possibly even enhanced synaptic density. Supplementation of G_M1_ was even able to more than compensate negative effects on neurite outgrowth, apoptosis and synaptophysin accumulations that were observed when culturing DRG neurons without NGF. Beneficial effects were most pronounced when DRG neurons were treated with both NGF and G_M1_. Additionally, G_M1_ supplementation lead to a significant increase in viability and neurite outgrowth of canine DRG neurons cultured under hypoxic conditions, which occur during spinal cord lesions caused by disc herniation, spinal stenosis, tumors, and spine trauma^61^. G_M1_ also increased the formation of multivescicular bodies indicating changes in cell metabolism. Moreover, G_M1_ might stimulate axonal transport mechanisms and increase the electrical excitability of these sensory neurons. These findings motivate further investigations upon the efficacy of ganglioside treatment in canine models of different traumatic, age-dependent and degenerative diseases of the nervous system.

## Materials and Methods

### Tissues used

DRGs of cervical, thoracic, and lumbar spinal cord segments from 19 healthy Beagle dogs (16 weeks to 2 years old) were used for cell culture experiments, transmission electron microscopy (TEM), and electrophysiology. The dogs were euthanized in the context of other studies, conducted in compliance with the law of animal welfare of Lower-Saxony and North Rhine-Westphalia, Germany and approved by Lower Saxony State Office for Consumer Protection and Food Safety, permission numbers: 33.9-42502-05-14A443, and 33.19-42502-05-16A044; and Ministry for Climate Protection, Environment, Agriculture, Conservation and Consumer Protection of the State of North Rhine-Westphalia, permission numbers: 501/A79, and T100525-3. In addition, cell culture experiments were also performed with DRGs of morphologically unchanged cervical, thoracic, and lumbar spinal cord segments obtained from one euthanized female adult dog (7 years old) during a routine necropsy at the Department of Pathology of the University of Veterinary Medicine Hannover. For the experiments in the hypoxia chamber, cryopreserved canine DRG neurons were used as previously described^14^ due to the lack of fresh tissue samples.

### Cell culture and immunofluorescence

Cell isolation was performed as previously described with slight modifications^128^. Briefly, DRG neurons were separated by enzymatic digestion for 30 min at 37 °C (25 ganglia per tube (BD Falcon round-bottom tube; 352059; BD Biosciences, Erembodegem, Belgium); 2 tubes per dog) in a mixture of type IV-S hyaluronidase (H3884; Sigma-Aldrich Chemie GmbH, Taufkirchen, Germany), type IV collagenase (C5138; Sigma-Aldrich) and type XI collagenase (C7657; Sigma-Aldrich) in a 0.2% solution (each enzyme) in 1x Hank’s Balanced Salt Solution (HBSS; Gibco^®^, Invitrogen GmbH, Darmstadt, Germany). After 30 min, type I trypsin (T8003; 0.2% solution; Sigma-Aldrich) was added followed by 30 min incubation at 37 °C. For mechanical dissociation successively narrowed flame-constricted Pasteur pipettes were used and DNase I (0.2%; Roche Diagnostics Deutschland GmbH, Mannheim, Germany) was added. Cell suspension was pelleted by centrifugation (5 min, 300 × g, 4 °C), and re-suspended in Dulbecco’s modified Eagle medium (DMEM; Gibco^®^, Invitrogen) with 10% fetal calf serum (FCS; Biochrom AG, Berlin, Germany) and 1% penicillin-streptomycin (PAA Laboratories GmbH, Pasching, Austria). The purification step included a two-step density gradient centrifugation (15 min, 450 × g, 4 °C) in 25% and 27% Percoll (GE Healthcare Europe GmbH, Freiburg, Germany) diluted in 1× HBSS. Finally, neurons were seeded in Sato’s medium (Bottenstein and Sato, 1979) with 1% bovine serum albumin (BSA; PAA Laboratories GmbH; Pasching, Austria) at a density of 70 neurons per well in 96 Half Area Well Microplates (CLS 3696; Corning^®^, Sigma-Aldrich) coated with poly-L-lysin (0.1 mg/ml; Sigma-Aldrich) and laminin (0.1 mg/ml; Becton Dickinson GmbH, Heidelberg, Germany).

The effects of gangliosides, NGF, and FGF2 on DRG neurons including their interactions were characterized in three steps. Firstly, in order to determine the concentration of G_M1_ ganglioside with maximum effects, DRG neurons were supplemented with 30 ng/ml human β nerve growth factor (NGF; 450-01; PeproTech GmbH, Hamburg, Germany) and 0, 10, 50, 80, 100, 150, 200, and 300 µM G_M1_ ganglioside (G7641; Sigma-Aldrich). The number of neuronal processes was determined 2 dps. Based on the results of this experiment all following experiments with G_M1_ ganglioside were performed with a concentration of 80 µM. Secondly, for analyzing the impact of NGF, FGF2, and G_M1_ alone and in combination on canine DRG neurons, preparations were cultured for 2 days with 6 different conditions: i) medium without supplements, ii) 30 ng/ml NGF (NGF), iii) 30 ng/ml FGF2 (100-18B; PeproTech GmbH, Hamburg, Germany, FGF), iv) 80 µM G_M1_ ganglioside (G_M1_); v) 30 ng/ml NGF and 80 µM G_M1_ ganglioside (NGF/G_M1_), and vi) 30 ng/ml FGF2 and 80 µM G_M1_ ganglioside (FGF/G_M1_). Finally, to investigate the effects of G_M2_ and G_M3_ supplementation and depletion of all cellular ganglioside subspecies on neurite outgrowth and neuronal survival, DRG neurons were cultured for 2 days with i) medium with 30 ng/ml NGF (control), ii) 30 ng/ml NGF and 200 µM glucosylceramide synthase inhibitor D-threo-PDMP (ab144052, Abcam, Berlin, Germany), iii) 30 ng/ml NGF and 80 µM G_M2_ (G8397; Sigma-Aldrich), and iv) 30 ng/ml NGF and 80 µM G_M3_ (G5642; Sigma-Aldrich). Gangliosides were sonicated after dissolving to avoid the formation of micells.

The first two sets of experiments were performed in triplicates, whereas quadruplicates were used in the third experiment. All experiments were analyzed using immunofluorescence as described^129^. Antibodies and dilutions used are shown in Table 1. The number of neuronal class III β-tubulin (βIII tubulin) or NF-positive processes per neuron were counted. The number of neurons with and without immunoreaction for Tau1, p75^NTR^, and cleaved caspase 3 was determined. Immunostainings for microtubule-associated protein (MAP) 2, nNF, and pNF were evaluated by counting the number of neurons with and without immunopositive processes to characterize the effects of NGF, FGF2, and G_M1_ ganglioside on neuronal cytoskeleton. In addition, due to the detection of accumulations of synaptophysin and of the axonal transport proteins dynein and kinesin in neuronal processes the number of neurons with and without respective accumulations in their processes was determined. Moreover, a Trypan blue dye exclusion assay (C.I. 23850; VWR International GmbH, Hannover, Germany) was used to investigate neuronal death in the third set of experiments. Finally, respective percentages were calculated and statistically evaluated.

**Table 1:** Antibodies used to characterize adult canine dorsal root ganglia neurons and non-neuronal cells by immunofluorescence.

Antigen	Antibody/Company	Dilution
**βIII tubulin** neuronal class III β tubulin	Covance; MRB-435P; rmAb	1:1000
**Cleaved caspase 3**	Cell Signaling; Asp175, 9961; rpAb	1:500
**Dynein**	Covance; MMS-400R; mmAb	1:500
**Kinesin-5A**	Sigma; K0889; rpAb	1:1000
**MAP2** (2a + 2b) Microtubule-associated protein 2	Sigma-Aldrich; clone AP-20; mmAb	1:500
**Neurofilament** Pan-neurofilament	Dako; clone 2F11; mmAb	1:100
**nNF** Non-phosphorylated neurofilament	Covance; SMI311; mmAb	1:1500
**pNF** Phosphorylated neurofilament	Covance; SMI312; mmAb	1:2000
**p75**^NTR^* Low affinity neurotrophin receptor	ATTC, clone HB8737; mmAb	1:2
**Synaptophysin**	Dako; clone SY38; mmAb	1:10
**Tau1**	Millipore; clone PC1C6M; mmAb	1:200

**mmAb** = mouse monoclonal antibody; **rmAb** = rabbit monoclonal antibody; **rpAb** = rabbit polyclonal antibody; *vital staining.

For lipid analysis, N1E-115 murine neuroblastoma cells (ATCC, CRL-2263) were differentiated using 1% DMSO for two days and cultured in either DMEM medium with 10% FCS, 1% penicillin-streptomycin and 1% sodium-pyruvate (S11-003; PAA Laboratories GmbH, Cölbe, Germany) containing 80 µM G_M1_ or without for an additional two days^130^. Thereafter, lipid rafts were isolated and analyzed by TLC using a previously published protocol with minor amendment^89,131^. Briefly, cells were washed in PBS, lysed in 1 ml 1% Triton X-100 in PBS using a 21 G syringe and rotated for 2 hours at 4 °C. Thereafter, cell debris was removed by centrifugation at 17,000 × g for 20 min at 4 °C, followed by a second centrifugation at 100,000 × g for 90 min at 4 °C. Lipids were isolated using a chloroform, methanol and water based method and separated by using TLC. The lipids were visualized using orcinol monohydrate staining (O1875-10G; Sigma-Aldrich) and compared to a standard run on the same TLC plate (TLC Silica gel 60 F254 plates, 1.05729.0001; Merck, Darmstadt, Germany). The limit of quantification was determined using three times the y-intercept and given as 230 ng and the limit of detection at 50 ng.

### Transmission electron microscopy

Light microscopical changes characterized by immunofluorescence were most prominent in DRG neurons supplemented with 30 ng/ml NGF and 80 µM G_M1_ ganglioside. To characterize the changes induced by G_M1_ ganglioside at the ultrastructural level, DRG neurons supplemented with 30 ng/ml NGF in combination or without G_M1_ ganglioside (80 µM) were compared using TEM. For this purpose, 2240 neurons/well were seeded on 6 well plates, fixed for 24 h in 2.5% glutaraldehyde solution, rinsed with 1% sodium cacodylate buffer (pH 7.2), post-fixed in 1% osmium tetroxide, and embedded in EPON 812 (Serva, Heidelberg, Germany). Sections were stained with lead citrate and uranyl acetate and investigated using an EM 10 C (Carl Zeiss Jena GmbH, Oberkochen, Germany) and a slow-scan 2K-CCD camera (TRS Tröndle, Moorenweis, Germany)^132^.

The number of multivesicular bodies in the soma of all neurons present in the investigated sections was determined (0 µM G_M1_: n = 16 neurons; 80 µM G_M1_: n = 18 neurons). In addition, the length of the neurites and the number of nodular enlargements with and without mitochondria were counted in all neurites (0 µM G_M1_: n = 17 neurites; 80 µM G_M1_: n = 27 neurites), that were present in the investigated sections. This was performed by using photos and analysis software (analySIS 3.1 software package; Soft Imaging system, Münster, Germany).

### Electrophysiology

To complete the analysis of G_M1_-induced effects on canine DRG neurons, functional changes of neurons supplemented with 30 ng/ml NGF in combination (G_M1_-treated) or without (non-treated) G_M1_ ganglioside (80 µM) were investigated using electrophysiology. Cultured DRG cells were placed on their coverslip under an upright BX51 WI Olympus microscope and continuously perfused with extracellular recording solution containing (in mM) NaCl 125, NaHCO_3_ 25, NaH_2_PO_4_ 1.25, KCl 2.5, D-Glucose 25, L-Ascorbic acid 0.4, Myo-Inositol 3, Na-pyruvate 2, MgCl_2_ 1, CaCl_2_ 2 at a pH 7.4 and was oxygenated with 95% O_2_ and 5% CO_2_. Electrophysiological recordings were carried out between 26–28 °C with an EPC 10/2 amplifier (HEKA, Lambrecht/Pfalz, Germany). Stimulus generation and presentation was controlled by the PatchMaster software. Cells were visualized with CCD-cameras (TILL-Imago VGA, Retiga 2000DC) controlled by TILLvisION imaging system (FEI Munich GmbH, Munich, Germany). In general, large DRG neurons were selected. Recordings were performed in whole-cell configuration using an intracellular solution containing (in mM) K-gluconate 145, KCl 4.5, HEPES 15, Mg-ATP 2, K-ATP 2, Na_2_-GTP 0.3, Na2-phosphocreatine 7, K-EGTA 0.5, Alexa488 0.05. Data were acquired with 20 kHz, and filtered by 3 Hz. Access resistance was compensated in voltage clamp mode before switching into current clamp, where bridge balance was set to 100%. For determining the input resistances and the cell capacitance by voltage clamp recordings all filters and the clamp were removed. Data was not corrected for the liquid junction potential of ~15 mV.

Cells were challenged with a −25 pA current injection of 120 ms length with 50 repetitions. The average voltage response to this hyperpolarization was used to determine membrane decay time constant by a mono-exponential fit. Using Ohm’s law the input resistance during steady state was calculated. To obtain a second estimate of the cells input resistance and to calculate the cells effective capacitance the average current of 20 repetitions of a 150 ms long −10 mV hyperpolarization was recorded in voltage clamp. The input resistance was again estimated following the Ohm’s law from the steady state current. The cell capacitance was determined from the area under the current transients for a time frame of three times the decay time constant. To probe for action potentials properties a 1 ms square current injection was applied and incremented by 100 pA. The first supra-threshold response to this current injection was used to analyze the action potential properties. Data analysis was carried out in IgorPro6 (Wavemetrics).

### Hypoxia chamber

In a first experiment, cryopreserved neurons were thawed according to a previously published protocol^14^ and seeded in two 96 Half Area Well Microplates at a density of 150 neurons per well in Sato’s medium containing 30 ng/ml NGF. Both titer plates contained one group of neurons treated with 80 µM G_M1_ and one group without G_M1_ supplementation. One of the two titer plates was directly transferred into a hypoxia glove-chamber (Coy Laboratory Products, Grass Lake, MI, USA), where it was cultured with 1% O_2_ (7 mm Hg, 5% CO_2_) for 6 days. The other titer plate was cultured under normoxia (21% O_2_; 5% CO_2_) for 6 days. The medium was changed on day 4 in both titer plates. After 6 days, immunofluorescence staining for cleaved caspase 3 and pan-neurofilament and a Trypan blue dye exclusion assay were performed.

Since cultivating canine DRG neurons under hypoxia for 6 days directly after seeding resulted in an almost complete absence of neurite-outgrowth, a second experiment was performed in order to evaluate the influence of G_M1_ treatment upon neurite outgrowth under hypoxic conditions. Cryopreserved neurons were again seeded in a 96 Half Area Well Microplate at a density of 150 neurons per well. Neurons were cultured under normoxia (21% O_2_; 5% CO_2_) for 6 days in Sato’s medium supplemented with 30 ng/ml NGF and a medium-change was performed on day 4. After 6 days, the neurons were transferred to the hypoxia glove-chamber (1% O_2_, 7 mm Hg, 5% CO_2_) to remain there for 48 hours according to a previously published protocol^133^. For the 48 hours under hypoxic conditions, the medium was changed again and one of the two groups of neurons on the titer plate was treated with 80 µM G_M1_, while the other group remained in Sato’s medium supplemented with NGF (control). Media used for this medium-change were pre-equilibrated in the hypoxia glove-chamber for 5 hours prior to treatment of the cells. On day 8 immunofluorescence staining for cleaved caspase 3 and pan-neurofilament and a Trypan blue dye exclusion assay were performed. All experiments were performed in quadruplicates and evaluated as described for the other cell culture experiments.

### Statistical analysis

Statistical analysis was performed using GraphPad software (Prism 6; GraphPad Software, Inc., La Jolla, CA, USA). Immunofluorescence data were evaluated using a one-way analysis of variance (ANOVA) followed by Dunnett’s multiple comparisons test (influence of G_M1_ on neurite outgrowth; comparison of supplemented groups versus control) or Tukey’s multiple comparisons test (influence of G_M1_, NGF, and FGF2 on protein expression; comparison of groups supplemented with G_M1_, NGF, and FGF2 versus control and comparison of groups supplemented with NGF/G_M1_ and FGF2/G_M1_ versus G_M1_, NGF, and FGF2 alone). TEM data (neurons supplemented with and without 80 µM G_M1_) were analyzed using Mann-Whitney tests (density of multivesicular bodies) and Fisher’s exact tests (nodular enlargements with and without mitochondria). Fisher’s exact tests were also used to analyze Trypan blue dye exclusion assays. Mean values are given in the description of the results, whereas figures also show single values. Electrophysiological data is shown as mean ± standard error of the mean (SEM) and assayed with a two-tailed unpaired t-test. Two-tailed unpaired t-tests were also used to analyze effects of G_M2_, G_M3_ and D-PDMP supplementation on neurite outgrowth and cleaved caspase 3 expression. *P* values < 0.05 were considered statistically significant.

## Supplementary information

Supplementary Information.

## Data Availability

The datasets generated and analyzed during the current study can be obtained from the corresponding author on reasonable request.
