# Supplementary material for: Neurotrophic effects of GM1 ganglioside, NGF, and FGF2 on canine dorsal root ganglia neurons in vitro
Source: Sci Rep. 2020 Mar 25;10:5380. doi: 10.1038/s41598-020-61852-z (PMC7096396; doi:10.1038/s41598-020-61852-z)

Supplementary Information

Neurotrophic effects of G_{M1} ganglioside, NGF, and FGF2 on canine dorsal root ganglia neurons *in vitro*

S. Schwarz, A. Lehmbecker, W. Tongtako, K. Hahn, Y. Wang, F. Felmy, I. Zdora, G. Brogden, K. Branitzki-Heinemann, M. von Köckritz-Blickwede, W. Baumgärtner, I. Gerhauser

Suppl. Fig. 1: Neuronal class III β tubulin, microtubule-binding protein (Tau1), microtubule-associated protein (MAP) 2, phosphorylated neurofilament (pNF), and non-phosphorylated neurofilament (nNF) expression in canine dorsal root ganglia neurons. The neurons were grown without growth factors or G_{M1} ganglioside (control) or supplemented with fibroblast growth factor 2 (FGF), nerve growth factor (NGF), G_{M1}, FGF and GM_1, or NGF and G_{M1} and stained 2 days post seeding. Shown are representative pictures of neurons supplemented with FGF, NGF, G_{M1}, or FGF and G_{M1}. Encircled areas mark Tau1 negative neurons. Note MAP2 positive processes of neurons (arrows). Bars = 50 µm.

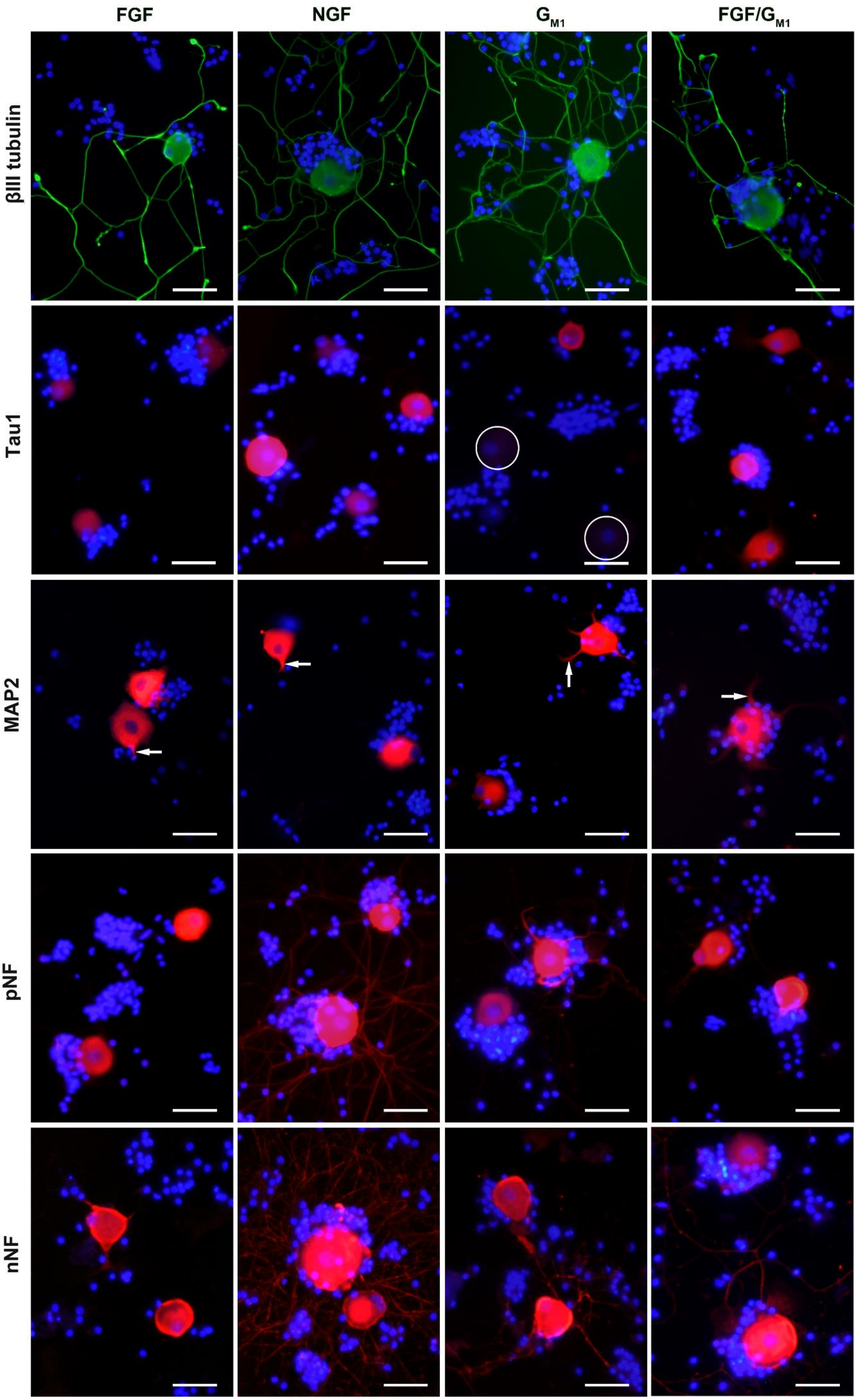

FGF
NGF
G M1
FGF/G M1
βIII tubulin
Tau1
MAP2
pNF
nNF

Suppl. Fig. 2: Neurofilament and cleaved caspase 3 expression in canine dorsal root ganglia neurons. The neurons were grown with nerve growth factor (NGF) alone (control) or supplemented with NGF and the glycosylceramide synthase inhibitor D-PDMP or NGF and gangliosides (G_{M2} or G_{M3}) and stained for neurofilaments (green) and cleaved caspase 3 (nNF, red) 2 days post seeding. Shown are representative pictures of neurons supplemented with NGF and D-PDMP, G_{M2} or G_{M3}. Note cleaved caspase 3 positive neurons (arrows) and cleaved caspase 3 negative neurons (arrowheads). Bars = 50 µm. The graphs show the number of neurofilament positive processes per neuron or the percentage of cleaved caspase 3 positive cells (single values of four wells with means). * $P < 0.05$.

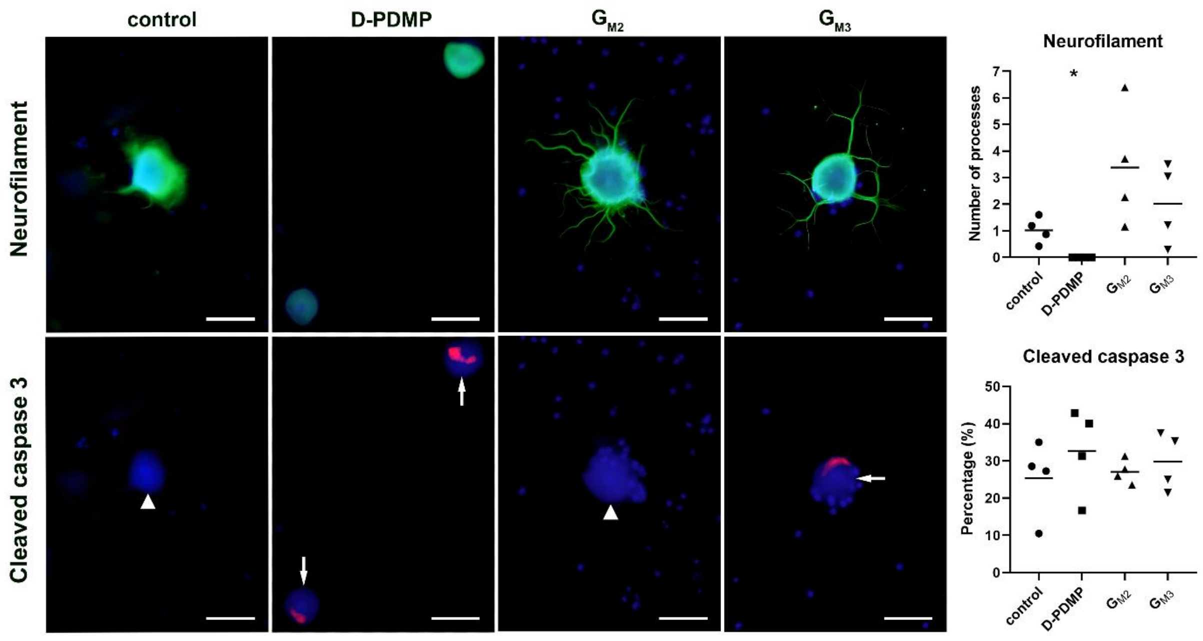

Suppl. Fig. 3: Trypan blue dye exclusion assay of canine dorsal root ganglia neurons. The neurons were grown with nerve growth factor (NGF) alone (control) or supplemented with NGF and the glycosylceramide synthase inhibitor D-PDMP or NGF and gangliosides (G_{M2} or G_{M3}). Trypan blue dye exclusion assay was performed 2 days post seeding. Shown are representative pictures of neurons supplemented with NGF and D-PDMP, G_{M2} or G_{M3}. Note positive neurons (arrows) and negative neurons (arrowheads). Bars = 50 µm. The graph shows the percentages of negative and positive cells for each condition. * P < 0.05.

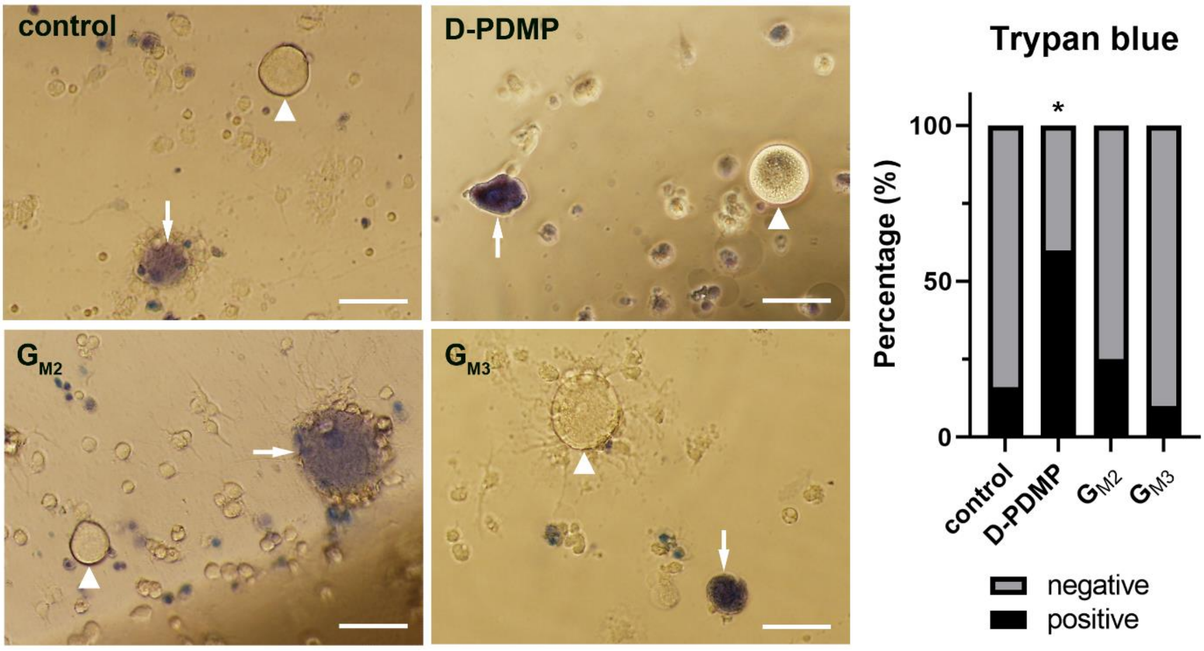

Suppl. Fig. 4: Synaptophysin, dynein, and kinesin expression in canine dorsal root ganglia neurons. The neurons were grown without growth factors or G_{M1} ganglioside (control) or supplemented with fibroblast growth factor 2 (FGF), nerve growth factor (NGF), G_{M1}, FGF and GM_1, or NGF and G_{M1} and stained 2 days post seeding. Shown are representative pictures of neurons supplemented with FGF, NGF, G_{M1}, or FGF and G_{M1}. Note accumulations of synaptophysin, dynein, and kinesin most likely within neuronal processes (arrows). Bars = 50 µm.

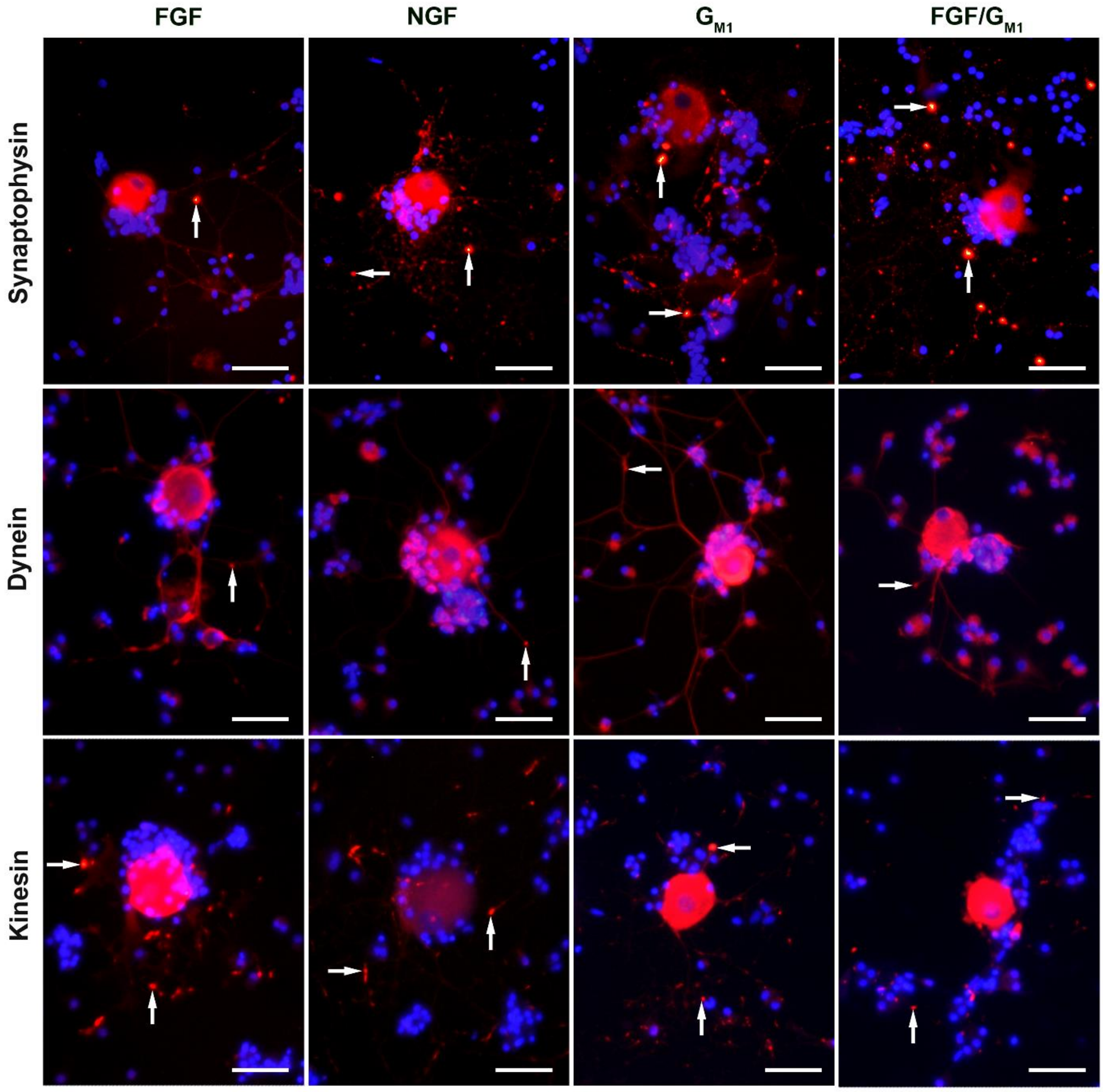

Suppl. Fig. 5: Expression of the p75 neurotrophin receptor ($p75^{NTR}$) in canine dorsal root ganglia neurons. The neurons were grown without growth factors or G_{M1} ganglioside (control) or supplemented with fibroblast growth factor 2 (FGF), nerve growth factor (NGF), G_{M1}, FGF and GM_1, or NGF and G_{M1} and stained for neuronal class III β tubulin (green) and $p75^{NTR}$ (red) 2 days post seeding. Shown are representative pictures of neurons supplemented with FGF, NGF or G_{M1}. Note $p75^{NTR}$ positive neurons (arrows) and $p75^{NTR}$ negative neurons (arrowheads). Bars = 50 µm.

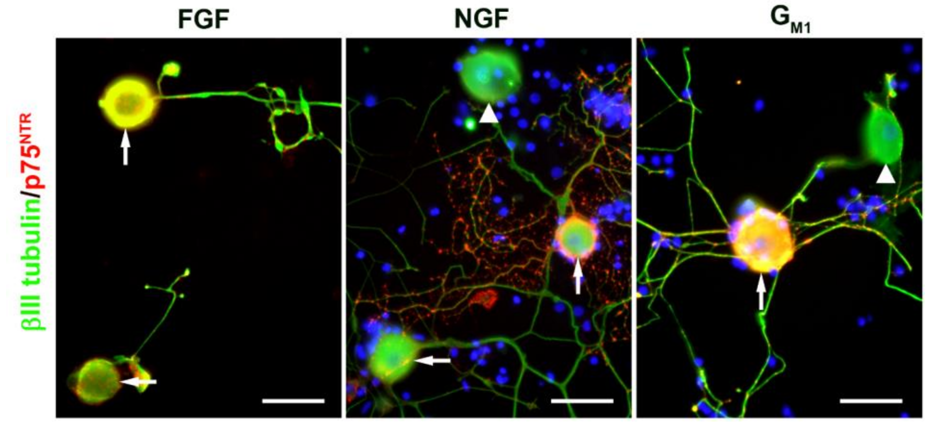

Suppl. Fig. 6: Cleaved caspase 3 expression in canine dorsal root ganglia neurons. The neurons were grown without growth factors or G_{M1} ganglioside (control) or supplemented with fibroblast growth factor 2 (FGF), nerve growth factor (NGF), G_{M1}, FGF and GM_1, or NGF and G_{M1} and stained for cleaved caspase 3 (green) and non-phosphorylated neurofilament (nNF, red) 2 days post seeding. Shown are representative pictures of neurons supplemented with FGF, NGF, G_{M1}, or FGF and G_{M1}. Note cleaved caspase 3 positive neuron (arrow) and cleaved caspase 3 negative neurons (arrowheads). Bars = 50 µm.

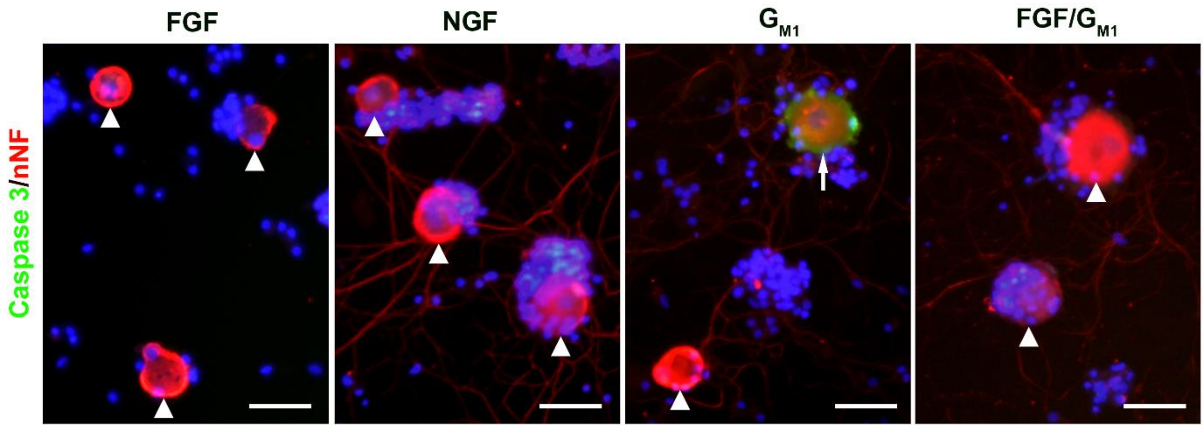

Supp. Fig. 7: Thin layer chromatography plate stained for glycolipids using orcinol. Murine neuroblastoma (N1E-115) cells were used to determine the cellular localization of exogenously administered G_{M1}. Detection of G_{M1} in the cell culture medium supplemented with G_{M1} but not in the medium without addition of G_{M1}. Presence of G_{M1} in the non-raft (NR) fraction of the sample treated with 80 mM G_{M1}. Confirmation of the presence of G_{M1} in the NR fraction through the addition of 500 ng G_{M1} to the same sample. Absence of G_{M1} in the lipid raft (LR) fraction from G_{M1} treated cells and in untreated cells.

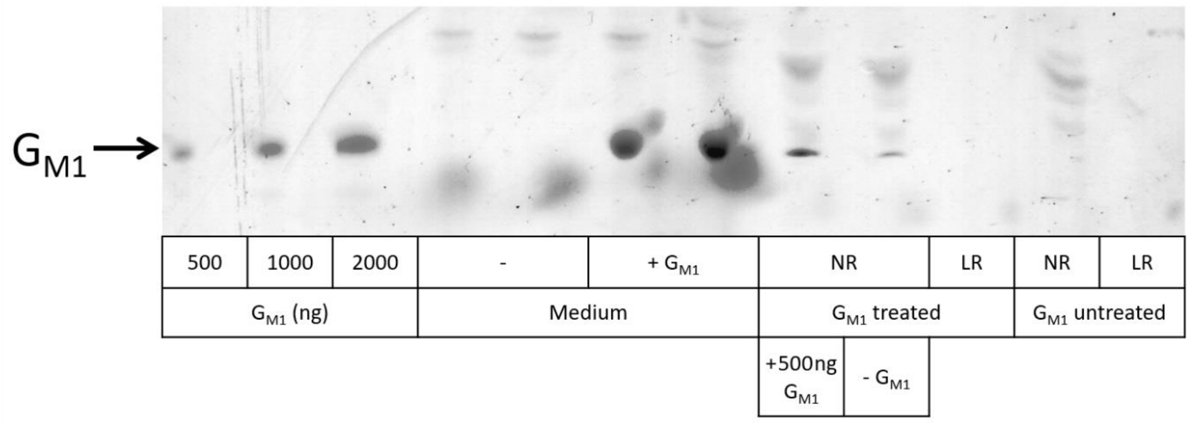

Suppl. Fig. 8: Influence of G_{M1} treatment upon cleaved caspase 3 expression of neurons cultured under hypoxia and normoxia. Neurons were grown in Sato's medium containing NGF under hypoxia (1 % O_2) or normoxia (21% O_2) with and without G_{M1} treatment for 6 days. G_{M1} treatment did not have a significant effect upon cleaved caspase 3 expression. Shown are representative pictures of neurons grown under hypoxia and normoxia with (G_{M1}) and without (control) G_{M1} supplementation. Note cleaved caspase 3 positive neurons (arrows) and cleaved caspase 3 negative neurons (arrowheads). Bars = 50 µm. The graph shows the percentage of cleaved caspase 3 positive neurons (single values of four wells with means). panNF = pan-neurofilament; Casp3 = cleaved caspase 3.

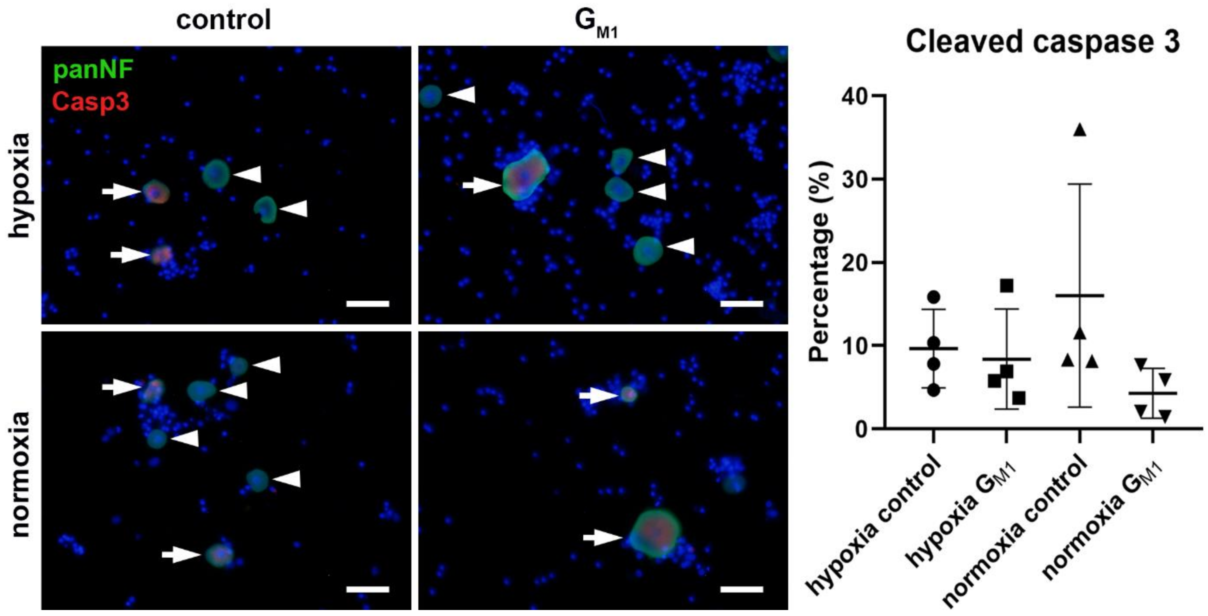

Suppl. Fig. 9: Trypan blue dye exclusion assay: Influence of G_{M1} treatment upon viability of neurons cultured for 6 days under normoxia followed by 48 hours of hypoxia. Neurons were cultured for 6 days under normoxia (21 % O_2) followed by a 48-hour period of hypoxia (1 % O_2), during which they either remained in Sato's medium supplemented with NGF (control) or received G_{M1} treatment (G_{M1}). In this experimental setup, there was no significant impact of G_{M1} treatment upon neuronal viability. Shown are representative images of neurons cultured under hypoxia with (G_{M1}) and without (control) G_{M1} supplementation. Note positive/dead (arrows) and negative/viable neurons (arrowheads). Bars = 50 μm. The graph shows the percentages of negative and positive cells for each condition.

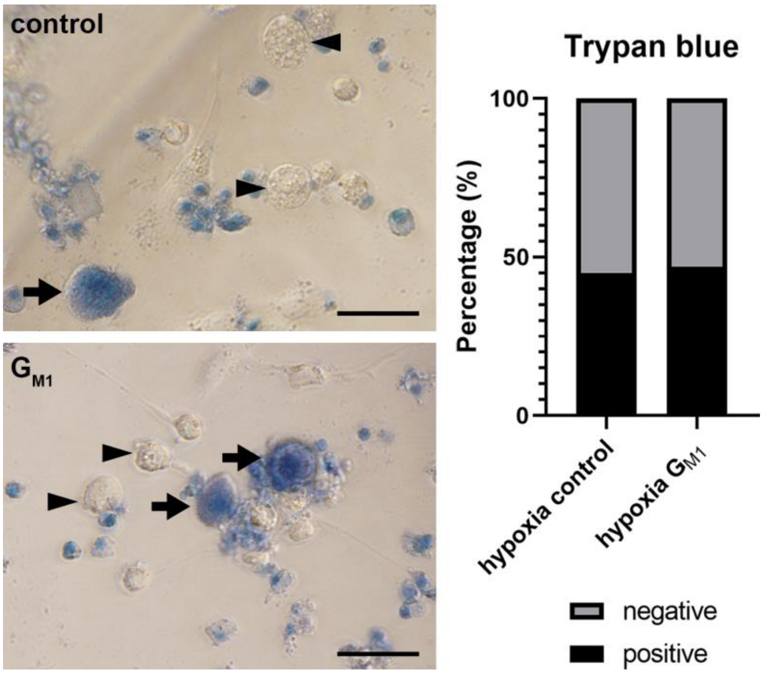

Suppl. Fig. 10: Influence of G_{M1} treatment upon cleaved caspase 3 expression of neurons cultured for 6 days under normoxia followed by 48 hours of hypoxia. Neurons were cultured for 6 days under normoxia (21 % O_2) followed by a 48-hour period of hypoxia (1 % O_2), during which they either remained in Sato's medium supplemented with NGF (control) or received G_{M1} treatment (G_{M1}). There was a reduction of cleaved caspase 3 expression in the group supplemented with G_{M1} compared to the control group, which did not reach significance. Shown are representative images of neurons cultured under hypoxia with (G_{M1}) and without (control) G_{M1} supplementation. Note cleaved caspase 3 positive neurons (arrows) and cleaved caspase 3 negative neurons (arrowheads). Bars = 50 µm. The graph shows the percentage of cleaved caspase 3 positive neurons (single values of four wells with means). panNF = pan-neurofilament; Casp3 = cleaved caspase 3.

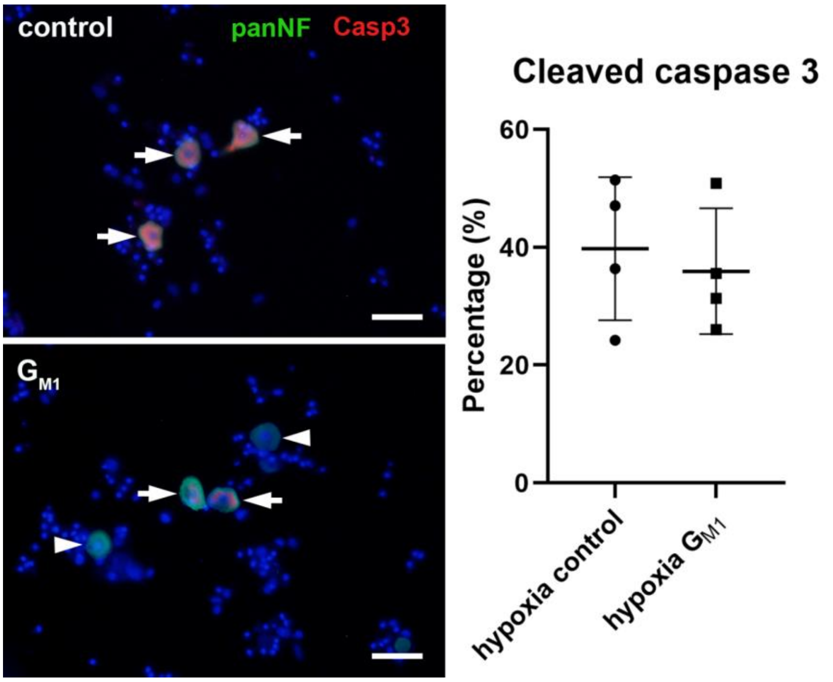

Supplement: Supplementary file 1 — Supplementary Information. [file 41598_2020_61852_MOESM1_ESM.pdf]
